# Systematic literature review and meta-analysis of the efficacy of artemisinin-based and quinine-based treatments for uncomplicated falciparum malaria in pregnancy: methodological challenges

**DOI:** 10.1186/s12936-017-2135-y

**Published:** 2017-12-13

**Authors:** Makoto Saito, Mary Ellen Gilder, François Nosten, Rose McGready, Philippe J. Guérin

**Affiliations:** 1 WorldWide Antimalarial Resistance Network (WWARN), Oxford, UK; 2 Centre for Tropical Medicine and Global Health, Nuffield Department of Medicine, University of Oxford, Old Road Campus, Roosevelt Drive, Oxford, OX3 7FZ UK; 3 Shoklo Malaria Research Unit (SMRU), Mahidol-Oxford Tropical Medicine Research Unit, Faculty of Tropical Medicine, Mahidol University, Mae Sot, Tak, Thailand

**Keywords:** Malaria, Pregnancy, Efficacy, Artemisinin, Quinine, Methodology, Review

## Abstract

**Background:**

There is no agreed standard method to assess the efficacy of anti-malarials for uncomplicated falciparum in pregnancy despite an increased risk of adverse outcomes for the mother and the fetus. The aim of this review is to present the currently available evidence from both observational and interventional cohort studies on anti-malarial efficacy in pregnancy and summarize the variability of assessment and reporting found in the review process.

**Methods:**

Efficacy methodology and assessment of artemisinin-based treatments (ABT) and quinine-based treatments (QBT) were reviewed systematically using seven databases and two clinical trial registries (protocol registration—PROSPERO: CRD42017054808). Pregnant women in all trimesters with parasitologically confirmed uncomplicated falciparum malaria were included irrespective of symptoms. This review attempted to re-calculate proportions of treatment success applying the same definition as the standard WHO methodology for non-pregnant populations. Aggregated data meta-analyses using data from randomized control trials (RCTs) comparing different treatments were performed by random effects model.

**Results:**

A total of 48 eligible efficacy studies were identified including 7279 treated *Plasmodium falciparum* episodes. While polymerase chain reaction (PCR) was used in 24 studies for differentiating recurrence, the assessment and reporting of treatment efficacy was heterogeneous. When the same definition could be applied, PCR-corrected treatment failure of ≥ 10% at any time points was observed in 3/30 ABT and 3/7 QBT arms. Ten RCTs compared different combinations of ABT but there was a maximum of two published RCTs with PCR-corrected outcomes for each comparison. Five RCTs compared ABT and QBT. Overall, the risk of treatment failure was significantly lower in ABT than in QBT (risk ratio 0.22, 95% confidence interval 0.07–0.63), although the actual drug combinations and outcome endpoints were different. First trimester women were included in 12 studies none of which were RCTs of ABT.

**Conclusions:**

Efficacy studies in pregnancy are not only limited in number but use varied methodological assessments. In five RCTs with comparable methodology, ABT resulted in higher efficacy than QBT in the second and third trimester of pregnancy. Individual patient data meta-analysis can include data from observational cohort studies and could overcome some of the limitations of the current assessment given the paucity of data in this vulnerable group.

**Electronic supplementary material:**

The online version of this article (10.1186/s12936-017-2135-y) contains supplementary material, which is available to authorized users.

## Background

Approximately 60% of all pregnancies worldwide take place in malaria endemic areas, leading to 125 million pregnant women at risk of malaria every year [1]. Malaria in pregnancy, regardless of whether it is clinically symptomatic or not, has been reported to be associated with a higher risk of preterm birth, low birth weight for gestational age, miscarriage, stillbirth and maternal anaemia [2–5]. These adverse outcomes lead to a higher risk of perinatal mortality and maternal mortality in areas with low or declining malaria prevalence compared to high transmission areas because of lower levels of premunition [6, 7].

In order to mitigate these adverse effects, efficacious treatments need to be clearly identified for pregnant women. However, several factors have limited the available evidence on anti-malarials efficacy during pregnancy. Pregnant women are usually excluded from randomized control trials (RCTs) of new anti-malarials mainly because of concerns about the safety for the fetus. Safety concerns were particularly critical for artemisinin derivatives, as fetal resorption was observed in animal studies [8–13]. Quinine-based treatment (QBT) is still recommended as the first-line treatment for uncomplicated falciparum malaria in the first trimester [14], despite limited clinical data on its safety in the first trimester [15, 16]. Quinine’s poor side effect profile and long treatment course of 5–7 days make it an undesirable choice for patients [15–17]. Recently, data from prospective observational cohort studies suggest that artemisinin use in the first trimester did not increase the risks of stillbirth or congenital abnormality compared to quinine [3, 18–21], bringing the question of comparative efficacy into center stage.

Efficacy of anti-malarial drugs for treating uncomplicated malaria in non-pregnant patients has traditionally been assessed over a fixed follow-up period set by the current World Health Organization (WHO) recommendations at 28–42 days [22]. However, this fixed period does not accommodate the pregnant condition as the placenta may become or remain parasitized (placental sequestration) after treatment completion [23]. There are currently no standard guidelines on parasitological efficacy studies in pregnancy [24].

With this in mind, this systematic literature review aims to update the currently available efficacy data of artemisinin-based treatments (ABT) and QBT from both observational and interventional cohort studies in all trimesters with uncomplicated falciparum malaria. Methodological challenges will also be summarized to improve future efficacy studies in pregnancy.

## Methods

A systematic literature review following PRISMA statement [25] was conducted to identify studies measuring the efficacy of ABT or QBT in pregnant women with parasitologically confirmed uncomplicated falciparum malaria, regardless of trimester or clinical symptoms. Seven databases (MEDLINE, Embase, Global Health, Cochrane Library, Scopus, Web of Science and LILACS) and two clinical trial registries (ICTRP and ClinicalTrial.gov) were used. This review is registered to PROSPERO (CRD42017054808), and the search terms and conditions are available in Additional file 1.

The search (conducted 9 July 2016–10 January 2017) combined five components: malaria; pregnancy; treatment or names of anti-malarial drugs; study design (interventional or observational cohort studies); and outcome types (efficacy) without limitation on publication year or language. Two reviewers (MS and MEG) assessed eligibility independently, and discrepant results were resolved by a second assessment.

Both interventional and observational cohort studies were included. Studies without any active follow-up in the first 28 days were excluded. Studies with fewer than ten pregnant women were excluded, as they could not be included in further meta-analyses. Systematic reviews on the anti-malarial treatment in pregnancy [15, 26–29] were checked for any other possible missing articles that should be included.

Uncomplicated *Plasmodium falciparum* malaria was defined as malaria infection without features of severe malaria [14]. Pregnancy was described by trimesters: the first as < 13 completed weeks, the second as from 14 weeks to 27 completed weeks, and the third from 28 weeks until delivery.

After screening, the following data were extracted: demographic information of study (year, country, study design, study drugs and eligibility criteria), availability of outcome assessment (clinical outcomes, parasitological outcomes) and the methodology of assessment of variables (definition of treatment success and statistical method). Information was sought from published articles, clinical trial registry and protocols if available. Missing information was supplemented by personal correspondence to authors of the original studies if possible. This review describes the methodology of assessment and reporting, and summarizes the reported efficacy results. Comparisons were made only if the same or similar assessment methods were used.

The WHO recommends that treatment failure and completion of follow-up without treatment failure (adequate clinical and parasitological response, ACPR) be used as efficacy endpoints in non-pregnant populations, and that results be expressed as the proportion of ACPR or the cumulative success using Kaplan–Meier survival analysis. WHO advises patients with polymerase chain reaction (PCR)-confirmed reinfection with *P. falciparum*, infection with other malaria species, or loss to follow-up to be censored on the day of these events in the survival analysis and to be excluded from the proportional ACPR analysis [22]. Patients without PCR results or with indeterminate PCR results are to be excluded from both PCR-corrected survival analysis and PCR-corrected proportional ACPR analysis.

For the comparability across studies, PCR-corrected proportional ACPR was recalculated using the WHO guideline for non-pregnant populations [22], with the exception of cases with non-falciparum malaria infection which could not be excluded without detailed individual patient data. When the number of patients with ACPR was unavailable, it was estimated from the presented results and included for reference. PCR-corrected ACPR of 90% was used as the cut-off value to judge whether the treatment was satisfactory or not [14]. The 95% confidence interval (CI) for the proportion was calculated by the Wilson method [30]. Random effects meta-analyses using aggregated results from RCTs were conducted if there were more than two RCTs. Heterogeneity was assessed using I^2^ [31]. If there was no treatment failure, a continuity correction was made by adding 0.5 for both treatment success group and failure group. The quality of data at both the study level and each outcome level was assessed using the GRADE system [32]. GRADEpro was used for making an evidence profile table [33]. For assessing publication bias, funnel plots of the proportions were drawn with log odds for the x-axis and study size (number of patients in each treatment arm) for the y-axis [34]. Asymmetry was judged visually, as formal statistical tests have not been developed for this method [34]. For odds ratios, Egger’s test was used for checking the asymmetry of funnel plots [35]. STATA MP 14.2 (Stata Corp, Texas, US) was used for the statistical analyses.

## Results

**Fig. 1:**
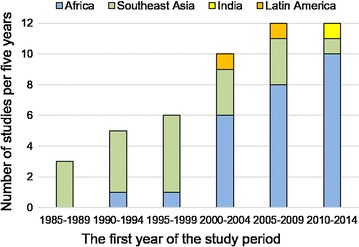
Number of studies on treatment efficacy of malaria in pregnancy per 5 years (1985–2016). The first year of study period was used for categorization. No studies were identified after 2015

A total of 48 study cohorts assessing treatment efficacy for uncomplicated falciparum malaria in pregnancy were identified (see Additional file 2) evaluating at least 7279 episodes of parasitologically confirmed uncomplicated falciparum. Studies based on the same cohort were considered as a single study. Forty-one studies were published, five presented at conferences, and two were registered on a public trials database but not yet published. Twenty-six studies (54%) were from sub-Saharan Africa, 19 (40%) from Southeast Asia, two (4%) from Latin America and one (2%) from India (Fig. 1). As of January 2017, all unpublished registered trials assessing anti-malarial efficacy in pregnancy were either completed (n = 2) or withdrawn (n = 3): two trials (NCT00331708, NCT01082731) were terminated because of slow recruitment, and the other (NCT01082718) was withdrawn before enrolment.

The study designs comprised 22 RCTs comparing two or more treatment regimens [17, 36–57], 10 pharmacokinetic (PK) studies including clinical outcome assessment [58–68], six single arm interventional studies [69–74] and 10 observational cohort studies [75–85] (Table 1 and Additional file 3). Blinding varied with one double-blind study [54] and one study using placebo but with unspecified blinding [17] (see Additional file 4). In seven studies, assessors and laboratory staff (n = 2) [45, 48] or only laboratory staff (e.g. microscopists) (n = 5) [44, 46, 50, 52, 57] were blinded.

**Table 1:** Summary of the methodology of assessing anti-malarial efficacy and its reporting

Name (references)	Design	Efficacy endpoint (day)	Malaria until delivery	Retreatment (previous drug)	Symptom	Supervision	PCR (molecular marker) for recurrence	Efficacy reported	Statistical method for summary	Correction by PCR
Naing [36]	RCT	7	INA	Not excluded	Symptomatic	Hospitalized	No	Parasite clearance time	Mean	No PCR
Harinasuta [37]	RCT	7	INA	No	INA	INA	No	Parasite clearance	Number	No PCR
Nosten [23]	RCT	28	Not followed	Not excluded	Both^a^	Full	No	Failure	Proportion	No PCR
Sowunmi [38]	RCT	14 for AM 28 for AM-MQ	INA	Yes (CHQ/SP)	Symptomatic	Full	No	Cure	Proportion	No PCR
Bounyasong [39]	RCT	42	Weekly	No	Symptomatic	Full	No recurrence	Failure	Number	No recurrence
McGready [40]	RCT	Delivery or 63	Weekly	Not excluded	Both^a^	Full	*msp*-*1*, *msp*-*2*, *glurp*	Cure	Cumulative success	Corrected only
McGready [41]	RCT	42	Weekly	Not excluded	Both^a^	Full	*msp*-*1*, *msp*-*2*, *glurp*	Cure	Cumulative success	Corrected only
McGready [42]	RCT	Delivery or 63	Weekly	No	Both^a^	Full	*msp*-*1*, *msp*-*2*, *glurp*	Cure	Cumulative success	Corrected only
Adam [43]	RCT	28	INA	Yes (CHQ)	Symptomatic	Full	*msp*-*1*, *msp*-*2*, *glurp*	Failure	Proportion	Corrected only
Kalilani [44]	RCT	28	Interval not specified	No	Both^a^	Full	*msp*-*1*	Failure	Proportion Survival analysis for comparison	Both corrected and uncorrected
McGready [45]	RCT	Delivery or 42	Weekly	28% recrudescence (Q, AS, DP, ASMQ)	Both^a^	Full	*msp*-*1*, *msp*-*2*, *glurp*	Cure	Cumulative success	Corrected only
Mutabingwa [46]	RCT	28	At delivery and 42 days postpartum	No	Both^a^	Full	*msp*-*2*	Failure	Proportion	Both corrected and uncorrected
Kaye [47]	RCT	28	Not followed	No	Symptomatic	First dose	No late failure	Failure	Proportion	No late failure
Piola [48]	RCT	Delivery or 42	Weekly	Not excluded	Both^a^	Full	*msp*-*1*, *msp*-*2*, *glurp*	Cure & failure	Proportion Log-rank test for comparison	Both corrected and uncorrected
Carmona-Fonseca [49]	RCT	28	Not followed	No	Symptomatic	INA	No recurrence	Cure	Proportion	No recurrence
D’Alessandro [50, 51]	RCT	63	Only at delivery	No	Both^a^	Full	*msp*-*1*, *msp*-*2*, *glurp*	Cure	Proportion Survival analysis for comparison	Both corrected and uncorrected
Osarfo [52]	RCT	42	Only at delivery	No	Asymptomatic	First dose	*msp*-*1*, *msp*-*2*, *glurp* (Failed)	Cure	Proportion	Uncorrected only
Onyamboko [53]	RCT	42	INA	No	INA	Full	INA	Cure	INA	Uncorrected only
Ukah [54]	RCT	28	Not followed	No	Symptomatic	INA	No	Cure	Proportion Log-rank test for comparison	No PCR
Iribhogbe [55]	RCT	28	Not followed	No	Symptomatic	INA	No	Failure	Proportion	No PCR
CTRI/2009/091/001055 [56]	RCT	63	INA	No	Both^a^	INA	Yes	Cure & failure	INA	INA
NCT01054248 [57]	RCT	Delivery or 63	Weekly	Not excluded	Both^a^	Full	Yes	Cure	Cumulative success	Both corrected and uncorrected
McGready [58]	PK study	Delivery or 42	Weekly	Yes (Q)	Both^a^	Full	*msp*-*1*, *msp*-*2*, *glurp*	Cure	Proportion	No recurrence
Adam [59]	PK study	63	Weekly (some)	No	INA	Full	Yes	Cure & failure	Number	Corrected only
Onyamboko [60]	PK study	28	INA	No	Asymptomatic	Full	No	Failure	Number	No PCR
McGready [61]	PK study	Delivery or 63	Weekly	Not excluded	Both^a^	Full	*msp*-*1*, *msp*-*2*, *glurp*	Cure	Cumulative success	Both corrected and uncorrected
Rijken [62]	PK study	Delivery or 63	Weekly	Two pregnant or non-pregnant women were self-treated (CHQ)	Symptomatic	Full	*msp*-*1*, *msp*-*2*, *glurp*	Cure	Cumulative success	Both corrected and uncorrected
Valea [63]	PK study	63	Passively	No	Both^a^	Full	*msp*-*1*, *msp*-*2*, *glurp*	Cure	Proportion	Both corrected and uncorrected
Juma [64]	PK study	28	INA	No	Symptomatic	Full	INA	INA	INA	INA
Mosha [65]	PK study	42	Not followed	No	Symptomatic	Full	*msp*-*2*	Cure & failure	Proportion	Uncorrected only
Nyunt [66]	PK study	42	Not followed	No	Symptomatic	INA	*msp*-*1*, *msp*-*2* and 4 microsatellites	Cure & failure	Proportion	Uncorrected only
Mutagonda [67, 68]	PK study	28	Not followed	No	INA	First dose and last dose	*msp*-*2*	Cure & failure	Proportion	Both corrected and uncorrected
Adam [69]	Single-arm	28	Every 2 weeks	Yes (CHQ)	Symptomatic	Full	No	Failure	Number	No PCR
Adam [70]	Single-arm	28	Every 2 weeks	Yes (CHQ and Q)	Symptomatic	Full	No recurrence	Cure	Proportion	No recurrence
Adegnika [71]	Single-arm	56	Only at delivery	No	Both^a^	First dose	No	Cure	Proportion	No PCR
Adam [72]	Single-arm	28	Every 2 weeks	No	Symptomatic	Full	No recurrence	Cure	Proportion	No recurrence
Ndiaye [73]	Single-arm	42	Only at delivery	No	Symptomatic	Full	No recurrence	Cure	Number	No recurrence
Iribhogbe [74]	Single-arm	28	Not followed	No	Symptomatic	INA	No	Failure	Proportion	No PCR
McGready [75]	Cohort	42	Weekly	68% retreatment (Q/MQ)	Both^a^	Full	No	Failure	Cumulative failure	No PCR
McGready [76]	Cohort	28	Weekly	45% retreatment (Q/MQ)	Both^a^	INA	No	Failure	Proportion	No PCR
McGready [77]	Cohort	42	Weekly	58% retreatment (Q/MQ)	Both^a^	Full	No	Failure	Proportion Cumulative failure	No PCR
Laochan [78]	Collation of studies	Delivery	Weekly	Some	Both^a^	INA	*msp*-*1*, *msp*-*2*, *glurp*	Time to recrudescence	Geometric mean	Corrected only
McGready [79]	Cohort	28	Weekly	Some	Both^a^	Recorded	No	Failure	Proportion	No PCR
McGready [80]	Cohort	42	Weekly	Yes (Q/QC/AS/AC)	Both^a^	Full	*msp*-*1*, *msp*-*2*, *glurp*	Cure	Proportion	No recurrence
Villegas [81]	Cohort	INA	INA	INA	Symptomatic	Full	INA	Cure	INA	INA
Rijken [82]	Cohort	63	Weekly	Yes (Q/QC/AS/AC)	Both^a^	Full	Yes	Cure	Cumulative success	Corrected only
Rulisa [83]	Cohort	56	INA	Some	Symptomatic	No	No	Cure & failure	Proportion	No PCR
Kalilani [84, 85]	Cohort	Delivery	Every 4 weeks	Not excluded	Both^a^	INA	6 microsatellite markers	Failure	Proportion	Both corrected and uncorrected

*AS* artesunate, *AC* artesunate + clindamycin, *AM* artemether, *CHQ* chloroquine, *DP* dihydroartemisinin–piperaquine, *MQ* mefloquine, *N* number of pregnant women included in the study, *INA* information not available, *PCR* polymerase chain reaction, *PK* pharmacokinetic study, *Q* quinine, *QC* quinine–clindamycin, *RCT* randomized control trial, *SP* sulfadoxine–pyrimethamine

^a^Both symptomatic and asymptomatic patients were included. Efficacy endpoint is the duration of follow-up for the primary endpoint. If pregnant women were followed up for malaria after the primary endpoint, the schedule is indicated in ‘Malaria until delivery’. Names of molecular markers are listed if they were specified

### Study drugs

Fourteen studies included women treated with QBT, 40 studies included ABT, and six studies included both. Altogether, 6244 and 1035 episodes were treated with ABT or QBT, respectively.

Quinine was administered at 30 mg/kg/day for 7 days in nine studies (64%, 9/14). The other five studies gave similar dosing with slight variations: one study administered for 7- or more-days depending on the clinical condition [39]; one study started with intravenous quinine until oral therapy was tolerated [36]; and one study compared standard dose with lower dose (20 mg/kg/day) for 7 days [43]; one study administered 30 mg/kg/day for 5 days [17]; one study gave 1800 mg/day for 7 days [37]. Artesunate was given at 10–16 mg/kg/course (600–900 mg/course) over 3–7 days except in two PK studies: one PK study administered 200 mg of artesunate once [60], and the other PK study administered total 28 mg/kg over 7 days (1 day intravenously and 6 days orally) [61]. Artesunate–amodiaquine (ASAQ) was given as a fixed dose combination in four studies (57%, 4/7) and as a non-fixed dose combination with 30 mg/kg of amodiaquine in three studies (43%, 3/7). Artesunate–mefloquine (ASMQ) was given as a fixed dose combination in four studies (36%, 4/11) and as a non-fixed dose combination with 25 mg/kg of mefloquine in six studies (55%, 6/11). Non-fixed dose combination of an unknown dose was assumed to be used in one study [81]. Artemether–lumefantrine (AL) was administered for 3 days (88%, 14/16) except two studies: one for 4 days [57] and the other for 5 days [53]. Supervision of treatments also varied (see Additional file 5).

Five RCTs compared QBT and ABT but only two of them used the same ABT (i.e. non-fixed dose combination ASMQ) [39–42, 48]. Ten RCTs compared different combinations of ABT [38, 45, 49, 50, 52–57] (see Additional file 6). Two RCTs compared different regimens of QBT [17, 43], two compared quinine versus non-ABT [36, 37] and three compared ABT versus non-ABT (e.g. sulfadoxine–pyrimethamine (SP) or chlorproguanil–dapsone) [44, 46, 47].

### Inclusion criteria

The demographic background of patients is summarized (see Additional file 7). Asymptomatic patients were included in 55% (12/22) of RCTs and 50% (13/26) of non-RCTs. Two of them included only asymptomatic women [52, 60]. Fifteen studies (31%, 15/48) intentionally included patients who failed previous treatment, but only three of them were RCTs [38, 43, 45].

Overall, twelve studies (25%, 12/48) included first trimester women (see Additional file 3), describing at least 599 parasitologically confirmed first-trimester malaria episodes treated with ABT (n = 108) or QBT (n = 491). Only one RCT intentionally included first trimester women; it was published in 1990 and compared quinine versus mefloquine [37].

In 26 studies from Africa, three studies gave two doses of intermittent preventive treatment in pregnancy (IPTp)-SP [54, 63, 66], and one study gave three doses [84]. One study reported the percentage of women who received IPTp [83]. IPTp-SP was not administered during the whole pregnancy in one study [48] and during the study period in two other studies [50, 52]. In one study, enrolled patients systematically received the study drug 4 weeks after the initial treatment instead of IPTp-SP [44].

### Assessment of treatment efficacy

#### Duration of follow-up

The primary endpoint for assessing anti-malarial efficacy varied: less than 28 days (n = 2), day 28 (n = 18), day 42 (n = 10), day 56 (n = 2), day 63 (n = 5) and until delivery (n = 10) (Table 1). The duration of follow-up was unclear for one conference abstract [81]. After the primary endpoint of 28–63 days, a further 14 studies continued assessing parasitaemia until delivery but with different schedules and five other studies did not continue but assessed parasitaemia only at delivery.

#### Methodology of reporting efficacy

For handling treatment failure, the majority of the studies (96%, 46/48) followed WHO standards for non-pregnant populations, except two studies which excluded cases who developed severe malaria after the treatment (i.e. early treatment failure) from the final result [40, 47].

Twenty-seven studies (56%, 27/48) showed only the proportional ACPR or the number of patients with ACPR (or failure). Ten studies [40–42, 45, 57, 61, 62, 75, 77, 82] derived cumulative success (or failure) by survival analysis instead of or in addition to proportional ACPR. Five other studies [44, 48, 50, 54, 68] used survival analysis to compare the different groups, but the proportional ACPR was presented as the point estimates. Two studies did not report ACPR but reported time to parasite clearance based on daily parasitaemia measurements [36] or time to recurrence [78]. Information was not available from four unpublished studies.

PCR was used in 24 studies (50%, 24/48) for differentiating recurrences (Table 1), although in one of the studies PCR results were considered not to be reliable because of technical problems [52]. Thirteen studies (27%, 13/48) used three standard molecular markers (i.e. *msp*-*1*, *msp*-*2* and *glurp*). Three of the studies adopted sequential genotyping strategy [48, 50, 63], while the remaining ten studies ran all three markers for all recurrences. Four studies (8%, 4/48) used one of the three markers [44, 46, 65, 68]. Two studies (4%, 2/48) used six microsatellite markers [66, 85]. Two published studies and two registered trials (8%, 4/48) did not specify the details of molecular markers [56, 57, 59, 82]. An additional six studies (13%, 6/48) did not observe any late treatment failure [39, 47, 49, 70, 72, 73], so a total of 29 (60%, 29/48) studies contained adequate information to obtain PCR-corrected results.

There was substantial heterogeneity in how reinfections and indeterminate PCR results were handled in deriving proportional ACPR. WHO recommends excluding these cases from proportional ACPR calculations in non-pregnant populations [22]. Reinfection was excluded except in two studies in which reinfections were included as treatment success [44, 50]. One study did not clearly report whether there were any reinfections [43]. Two studies excluded indeterminate outcomes from the calculation [48, 50], and one of them also presented results with imputation for indeterminate cases for reference [50]. Two studies using survival analysis regarded indeterminate results as recrudescence as the worst case scenario [40, 82]. One study did not explain how they dealt with missing PCR results [46]. For proportional ACPR, no studies clearly excluded patients infected with other species of malaria before recurrence of falciparum, as recommended by the WHO non-pregnant guideline. Only one study presented results of a model which censored patients infected with *Plasmodium vivax* before recurrence of *P. falciparum*, and this result was provided as additional information and not the primary analysis (which did not account for *P. vivax* infection) [45].

Gametocyte carriage is summarized in Additional file 8. Interruption of treatment and associated adverse symptoms are summarized in Additional file 5.

#### Fever and parasite clearance

Fever was assessed daily or more frequently in 27 studies (56%, 27/48) and time to fever clearance was reported in 12 studies (25%, 12/48). Prevalence of clearance of fever at fixed points (on day 2, 3 or 4) was reported in other 12 studies (25%, 12/48). Blood smear was assessed for parasitaemia daily or more frequently at least for the first 3 days in 30 studies (63%, 30/48) and time to parasite clearance was reported in 16 studies (33%, 16/48). The proportion of women who cleared parasitaemia at fixed points (i.e. by 12 h, on day 1, 2 or 3) was reported in another 14 studies (29%, 14/48).

The variability of reporting parasite and fever clearance limited comparisons across studies. In five RCTs comparing ABT and QBT, three and five RCTs reported the comparison of fever clearance and parasite clearance, respectively. One RCT reported that the mean time to fever clearance was significantly shorter by ASMQ than quinine (4.47 days vs 8.04 days, p < 0.001), although this time was from the appearance of fever according to the patients’ history rather than from the treatment [39]. Another two studies reported no difference in the proportion of febrile patients or the proportion of fever clearance by day 2 or 3 between ABT and QBT [42, 48]. On the other hand, all five RCTs reported faster parasite clearance by ABT than by QBT. The mean or median time to parasite clearance was shorter by ABT than by quinine in two RCTs [39, 42]. Similarly, the proportion of parasite clearance by 48 h [40, 41] and negative parasitaemia on day 2 [48] were higher with ABT than QBT, although this difference was not observed on day 3 [48]. 

Between different ACTs, lower parasite clearance on day 1 was reported with AL compared to ASAQ, ASMQ and dihydroartemisinin–piperaquine (DP), although this was not significant on day 2 [50]. Other two RCTs also showed no difference in parasite clearance on day 3 between AL and ASAQ [54, 55]. The median or mean time to fever and parasite clearance were not different between artesunate monotherapy and AL [45], and between intramuscular artemether with or without mefloquine, respectively [38].

#### Summary of reported ACPR

**Fig. 2:**
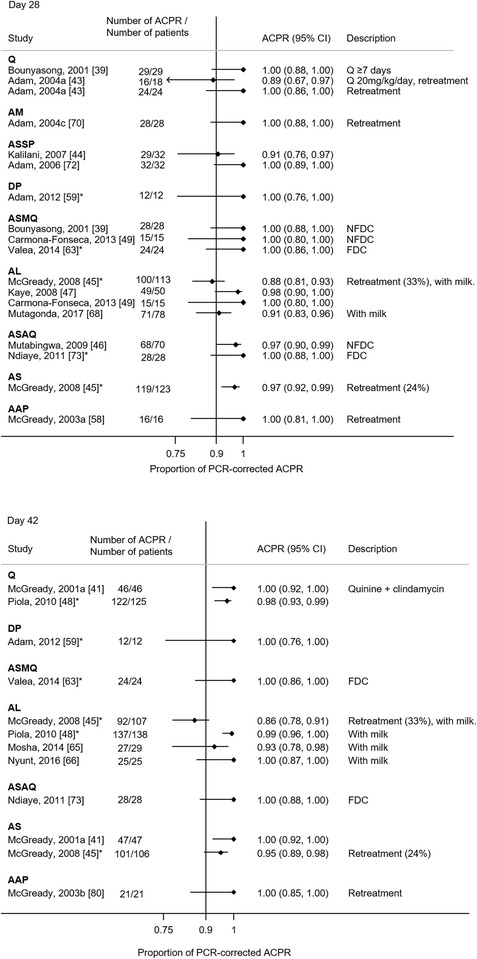
The PCR-corrected proportional adequate clinical and parasitological response (ACPR) for each study. ACPR are shown by treatment group and the duration of follow-up (i.e. day 28, 42, 63 and at delivery) with 95% confidence intervals. *ACPR at later follow-up day is available. *AAP* artesunate + atovaquone–proguanil, *AC* artesunate + clindamycin, *AL* artemether–lumefantrine, *AM* artemether, *AS* artesunate, *AQ* amodiaquine, *DP*: dihydroartemisinin–piperaquine, *FDC*: fixed dose combination, *MQ* mefloquine, *NFDC* non-fixed dose combination, *Q* quinine, *QC* quinine + clindamycin, *SP* sulfadoxine–pyrimethamine

**Fig. 3:**
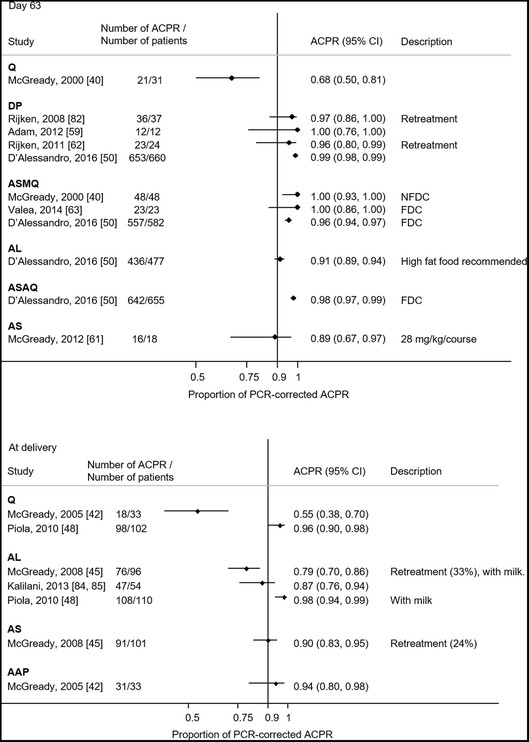
The PCR-corrected proportional adequate clinical and parasitological response (ACPR) for each study (continued). ACPR are shown by treatment group and the duration of follow-up (i.e. day 28, 42, 63 and at delivery) with 95% confidence intervals. *AAP* artesunate + atovaquone–proguanil, *AC* artesunate + clindamycin, *AL* artemether–lumefantrine, *AM* artemether, *AS* artesunate, *AQ* amodiaquine, *DP*: dihydroartemisinin–piperaquine, *FDC*: fixed dose combination, *MQ* mefloquine, *NFDC* non-fixed dose combination, *Q* quinine, *QC* quinine + clindamycin, *SP* sulfadoxine–pyrimethamine

PCR-corrected treatment failure of ≥ 10% at any time points was observed in 3 (10%, 3/30) ABT arms and 3 (43%, 3/7) QBT arms (Figs. 2, 3). On day 28, two study arms had PCR-corrected treatment failure of ≥ 10%. One of them used a lower dose quinine (10 mg/kg twice daily rather than thrice daily) [43]. In the other study which reported the low efficacy of AL, 33% of the enrolled patients were under retreatment, having failed previous treatment [45]. If retreatments were excluded, the cumulative success of AL on day 42 was > 90% [45]. Other study arms with PCR-corrected treatment failure of ≥ 10% were observed on day 63 (one quinine arm [40] and one artesunate monotherapy arm [61]) and until delivery (one quinine arm [42] and one AL arm [85]). The funnel plots for PCR-corrected ACPR on ABT and QBT did not show apparent asymmetry (see Additional file 9).

PCR-uncorrected ACPR was available in 14 QBT arms and 47 ABT arms (see Additional file 10). PCR-uncorrected ACPR at different time points was available in some studies, and if it was not reported on day 28 but was > 80% on day 42, 63 or at delivery, it was assumed to be > 80% on day 28. Although different levels of the risk of reinfection due to the time and location complicate comparison between studies, at least 50% (7/14) of QBT arms showed ≤ 80% protection within 28 days while at least 79% (37/47) of ABT arms showed > 80% protection within 28 days. Two ABT arms reporting ≤ 80% protection within 28 days were both conducted in high endemic areas and used AL or artesunate + SP [44, 74]. PCR-uncorrected ACPR on day 28 was not available but it was ≤ 80% after day 28 in other eight arms from six studies [45, 50, 61, 75, 77, 82]. ABT went on to provide protection in > 80% of patients at 42 days in at least 73% (22/30) of the study arms which followed for 42 days or longer.

#### PCR-corrected ACPR comparing different treatments

**Fig. 4:**
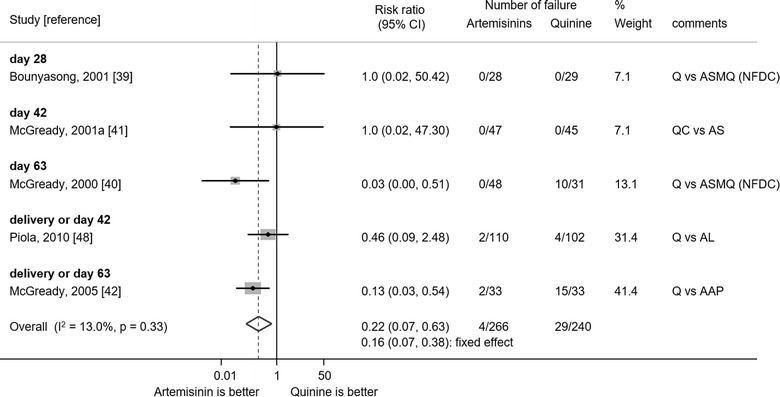
Meta-analysis of risk of PCR-corrected treatment failure comparing quinine-based treatment and artemisinin-based treatment. The outcome of longest duration of follow-up was used. Random effects model was used for meta-analyses. Continuity correction was made for two studies without treatment failure by adding 0.5. *AAP* artesunate–atovaquone–proguanil, *AL* artemether–lumefantrine, *AS* artesunate, *ASMQ* artesunate–mefloquine, *CI* confidence interval, *Q* quinine, *QC* quinine-clindamycin, *NFDC* non-fixed dose combination

Overall, the risk of treatment failure in the five RCTs available for meta-analysis was significantly lower in patients treated with ABT compared to QBT (risk ratio 0.22, 95% confidence interval 0.07–0.63) (Fig. 4), although the compared treatments and methodologies differed (see Additional file 11). This increased risk of failure with QBT was prominent when the patients were followed up longer. There was no evidence for asymmetry of the funnel plot suggesting publication bias (p = 0.7) (see Additional file 12). None of these five RCTs included pregnant women in the first trimester.

The limited number of available studies with PCR-corrected outcome comparing different ABTs precluded aggregated data meta-analysis. One RCT reported that PCR-corrected ACPR on day 63 was lower for AL than DP, ASAQ and ASMQ, although the absolute difference was < 5% [50]. Lower efficacy of AL than artesunate for 7 days was also reported when used for treating recrudescent infections [45].

#### Time to recrudescence

Recrudescence in pregnancy occurred after day 28 or 42, the fixed follow-up periods recommended by WHO for non-pregnant populations. In one study, one-third of the recrudescence following AL or artesunate monotherapy occurred after day 42 [45], and four studies reported recrudescence after 100 days with a maximum of 138 days [42, 44, 48, 78]. These long intervals were reported from both sub-Saharan Africa and Southeast Asia.

#### Risk factors for recrudescence in pregnancy

Baseline characteristics were assessed as risk factors of parasite clearance (n = 1) [36], recrudescence (n = 2) [51, 65] and recurrence (n = 3) [52, 67, 71]. One study reported gestation (early second trimester) to be associated with faster parasite clearance than late second trimester but not fever clearance [36]. Gravidity was not associated with parasite or fever clearance [36]. 

For recrudescence, higher baseline parasitaemia and younger maternal age were associated with higher risk in one study [51], while the other study did not find significant association with parasitaemia [65]. Gestational age (n = 2) [51, 65], gravidity (n = 1) [51], haemoglobin concentration (n = 1) [51], body mass index (n = 1) [65] and symptomatic infection (n = 1) [51] were not significant risk factors of recrudescence. 

For recurrence, higher baseline parasitaemia [71], younger age [71] and lower haemoglobin concentration [67] each were associated with higher risk in one of the studies, while the other two studies did not show the associations with parasitaemia [52, 67], age [52, 67] or baseline haemoglobin concentration [52, 71]. Lower body weight was shown to be associated with higher risk of recurrence [71]. Gestational age (n = 3) [52, 67, 71], gravidity (n = 3) [52, 67, 71], parity (n = 1) [71], body temperature (n = 1) [71] were not associated with the risk of recurrence.

The risk of recrudescence was higher when the drugs were used for retreatment (artemisinins [77] and quinine [76]) or recrudescence (AL) [45] than for novel infection.

#### Placental malaria

Placental malaria was assessed in 14 studies (29%, 14/48) in various ways (see Additional file 13). Placental histopathology is the gold standard and was examined in at least six studies [39, 44, 48, 50, 57, 84], but the reporting and interpretation of the results varied with at least four different definitions [86–89]. The prevalence of placental malaria after treatment ranged from < 10% [45, 48, 62, 80] to 44.8% [44], and was not different among different ABTs [50, 52] or between AL and quinine [48].

#### Congenital malaria

The peripheral blood of the newborn was assessed for malaria in six studies, of which three assessed all newborns systematically at delivery and reported the results [38, 45, 48]. Another two studies reported congenital malaria as a possible reason for neonatal deaths but did not report whether they tested for congenital malaria systematically [40, 42]. One further study assessed congenital malaria but did not report the results [63]. No studies specified further parasitological monitoring of infants for congenital malaria diagnosed after the perinatal period.

## Discussion

This review revealed the variability of study design, drugs, treatment regimens for the same drug, inclusion criteria, determination of parasitological efficacy, follow-up duration and detection of parasites in mother, placenta and newborn at delivery. The design of studies to assess the efficacy of anti-malarials during pregnancy is not standardized, and investigators are using varied adapted versions of the protocol for the non-pregnant populations currently recommended by WHO [22].

The efficacy of ABT was generally satisfactory with ACPR of > 90%, although aggregated efficacy results are limited by heterogeneous methods as well as differences in patient immunity, background, symptoms, time and study site. If aggregated results are used, only a maximum of two RCTs was available for each comparison. Slightly lower efficacy of AL than other ABTs was reported in two studies [45, 50], and this may be explained by differences in the pharmacokinetics and pharmacodynamics of lumefantrine in pregnant women [45, 48, 66, 67, 90–93]. On the Thailand–Myanmar border, the proportion of PCR-corrected ACPR of AL was markedly low in a study of pregnant women (< 85%) [45] and not comparable to that (> 95%) reported from studies in non-pregnant populations [94, 95]. Monitoring of efficacy in pregnancy remains a useful assessment in its own right. Optimal dosing for pregnant women should be sought and extending treatment course can be an option to improve the efficacy [96]. The results of two studies assessing longer duration and higher doses of AL [53, 57] are awaited.

The available data implied QBT has two major but not unexpected drawbacks compared to ABT (see Additional file 11): lower treatment efficacy and lower adherence. In at least 50% (7/14) of the study arms, one in five pregnant women or more suffered another episode of malaria parasitaemia (either recrudescence or reinfection) within 28 days after treatment by quinine. Although these studies were conducted mostly in Southeast Asia, the risk of recurrence of malaria is expected to be even higher in the high endemic areas in sub-Saharan Africa considering the short half-life of quinine. This finding is important as repetitive malaria infections during pregnancy increase adverse effects of miscarriage, small for gestational age and preterm birth cumulatively [18, 97]. Adherence to a 7-day treatment outside of a clinical trial context is difficult for pregnant women due to the common side effect of tinnitus, which is particularly intolerable in the first 4 months of pregnancy when morning sickness peaks [17]. Adding clindamycin to quinine might provide satisfactory efficacy equivalent to ABT, but it will not overcome the poor adherence.

Several components of efficacy evaluation should be standardized for future studies. Duration of the follow-up period needs to be optimized for pregnant women considering physiological variations related to pregnancy [15, 23, 40, 78]: placenta sequestration [98]; different drug metabolism and distribution [99, 100]; and altered immunity profile [98]. The current WHO recommendation of follow-up for 28–42 days [22] is based on the fact that the proportion of recrudescence after 28–42 days is negligible in the non-pregnant populations [101]. However, recrudescence in pregnancy was relatively commonly observed after 42 days, demonstrating the need for prolonged follow-up in pregnancy studies. In the small number of studies that have collected relevant data, recrudescence even after 100 days has been reported in pregnant women both in Africa [44, 48, 102] and Southeast Asia [42, 78]. The duration of parasitological follow-up after delivery also needs to be considered when delivery occurs before the end of the recommended follow-up.

Two problems were encountered with proportional ACPR for pregnancy studies. The lack of a standard led to inconsistent handling of reinfections in proportional ACPR calculations making comparisons across studies difficult. In non-pregnant populations, WHO recommends excluding those with reinfection or indeterminate PCR results from the calculation of PCR-corrected proportional ACPR [22]. However, higher proportions of reinfection and lost-to-follow-up are anticipated if longer follow-up is recommended for pregnant women. If proportional ACPR is calculated, much information will be lost. Survival analysis with censorship of these cases is likely to be a preferable approach for pregnancy studies [103].

Data on efficacy in the first trimester are limited to information from observational studies, precluding comparison of the outcomes using aggregated results. In addition, the published results were rarely presented separately by trimester, and the methods and quality of gestational age assessment were limited [104].

Finally, and importantly, it needs to be emphasized that pregnant women may experience several episodes of malaria during pregnancy. The relationship between placental malaria, which is a direct consequence of malaria infection, and treatment outcomes for each malaria episode is not fully understood and may be heavily biased by gestational age at infection [105]. In this context, PCR-uncorrected ACPR, in which the half-life of drugs will play a key role, becomes an important indicator for choosing treatment options especially in high endemic settings [51]. Efficacy cannot be discussed separately from pregnancy outcomes as drugs may affect the fetus. Ideally, the safety of drugs should be assessed and reported within efficacy studies, using a standard which has been reviewed elsewhere [106]. Inclusion rather than exclusion of pregnant women into routine therapeutic efficacy studies could be a key change to address the paucity of data in the future when drugs have a clean teratogenicity profile. This would also permit comparison of efficacy and drug levels in pregnant and non-pregnant females. Recommendations specific to the determination of anti-malarial efficacy of uncomplicated *P. falciparum* infection in pregnancy beyond the WHO guideline for non-pregnant populations are listed in Table 2.

**Table 2:** Recommendations to determine antimalarial efficacy in uncomplicated *P. falciparum* infection in pregnancy (beyond current WHO standards for non-pregnant patients)

Report the following	
Gestational age	
Gestational age in weeks	
Method of gestational age estimation and when it was obtained	
The proportion of pregnancies with different methods of gestational age estimation (optional)	
Quality control measures (desirable)	
Parity and gravidity	
Parity and gravidity	
Duration of follow-up	
Pragmatically at least adhere to the WHO guidelines for reporting outcomes on 28–42 days (optimal recommendations being likely to emerge from individual patient data analysis)	
Continue parasitological follow-up until delivery	
Record all episodes of *P. falciparum* and non-falciparum malaria	
Other antimalarials	
Document the type, date of administration and supervision (or self-taken) of IPTp	
Document the type, date of administration and supervision (or self-taken) of cotrimoxazole	
In the context of a RCT supervised treatment, treat parasite reappearance in each arm with the same efficacious regimen which should be different to the primary treatment (and preferably given under supervision)	
Placental malaria and congenital malaria	
Placental malaria and congenital malaria should be assessed as part of assessment of efficacy (desirable)	
PCR genotyping should be assessed for placental and congenital malaria and compared to the previous malaria infections during the pregnancy (desirable)	

The assessment of anti-malarial efficacy presented here is limited by the paucity of data, the heterogeneity of studies, and the constraints of aggregated data meta-analysis. With no new RCTs on efficacy in pregnancy notified in trial registries, the treatment episodes in the cohort of studies described here will be the only data available in the near future to describe the efficacy of anti-malarials in pregnancy. Because of the paucity of studies, different methodologies and backgrounds (regional endemicity, seasonality of infection) of patients, conventional meta-analysis using aggregated data will not allow an optimal assessment of the findings. Reviews attempting aggregated data meta-analysis, including this work, should be interpreted with caution considering the different endpoints and drugs used. In addition, RCTs comparing ABT and QBT were mostly conducted in Southeast Asia. Though the conclusion of the comparison between ABT and QBT is unlikely to change considering the large magnitude of the effect, assessing smaller differences between ABTs would be valuable and cannot be effectively done by aggregated data meta-analysis. Individual patient data meta-analyses offer a better methodological approach to summarize the currently available data, integrating the data from non-comparative studies. A new study group at the WorldWide Antimalarial Resistance Network [107] is proposed to tackle these issues by conducting individual patient data meta-analyses.

## Conclusions

Although this and other reviews of ABTs and QBTs for malaria in pregnancy suggest ABTs are superior, further aggregated meta-analysis was hampered by inconsistencies in measurement and reporting. A standard framework for anti-malarial efficacy studies in pregnancy is warranted and will be a foundation for research with more comparable and reliable outputs.

## Additional files

**Additional file 1.** Search terms for literature review.

**Additional file 2.** PRISMA flowchart.

**Additional file 3.** Summary of the study design and reported outcomes.

**Additional file 4.** Summary of the quality of studies.

**Additional file 5.** Summary of treatment administration and interruption.

**Additional file 6.** The number of study arms with PCR-corrected outcomes for each comparison of drugs.

**Additional file 7.** Background information of patients at inclusion.

**Additional file 8.** Summary of gametocyte carriage.

**Additional file 9.** Funnel plots by quinine-based and artemisinin-based treatments.

**Additional file 10.** The PCR-uncorrected proportional adequate clinical and parasitological response (ACPR).

**Additional file 11.** Summary of evidence profile comparing artemisinin-based and quinine-based treatment.

**Additional file 12.** Funnel plot of odds ratio of PCR-corrected treatment failure comparing quinine-based and artemisinin-based treatments.

**Additional file 13.** The methodology of assessing placental malaria.

## Abbreviations

ABT: artemisinin-based treatments
AC: artesunate + clindamycin
ACPR: adequate clinical and parasitological response
AL: artemether–lumefantrine
ASAQ: artesunate–amodiaquine
ASMQ: artesunate–mefloquine
CI: confidence interval
DP: dihydroartemisinin–piperaquine
IPTp: intermittent preventive treatment in pregnancy
PCR: polymerase chain reaction
PK: pharmacokinetic
QBT: quinine-based treatments
QC: quinine + clindamycin
RCT: randomized control trial
SP: sulfadoxine–pyrimethamine
WHO: World Health Organization
