# Supplementary material for: Systematic literature review and meta-analysis of the efficacy of artemisinin-based and quinine-based treatments for uncomplicated falciparum malaria in pregnancy: methodological challenges
Source: Malar J. 2017 Dec 13;16:488. doi: 10.1186/s12936-017-2135-y (PMC5729448; doi:10.1186/s12936-017-2135-y)
Supplement: Supplementary file 1 — Additional file 1. Search terms for literature review. [file 12936_2017_2135_MOESM1_ESM.pdf]

Additional file 1. Search terms for literature review

The terms used for literature search

(malaria or Plasmodium).m_titl.

"pregnan*".m_titl.

(treatment or therap* or antimalaria* or anti-malaria* or artemisinin* or artesunate* or dihydroartemisinin* or artemether* or chloroquine* or mefloquine* or amodiaquine* or quinine* or piperaquine* or lumefantrine* or atovaquone* or proguanil* or sulfadoxine* or pyrimethamine* or azithromycin* or clindamycin*).ab,kf,kw,ti.

(trial or cohort or prospective or follow or intervention or random* or pharmaco*).ab,kf,kw,ti.

(efficac* or success or failure or recrudescence or recurren* or reappear* or response or outcome or effect* or clearance or cure or dose or dosage or surviv*).ab,kf,kw,ti.

Depending on the number of conditions allowed to use for the search, I used only condition 1 and 2 for ClinicalTrial.gov, 1, 2 and 3 for ICTRP and LILACS.
