# Supplementary material for: Systematic literature review and meta-analysis of the efficacy of artemisinin-based and quinine-based treatments for uncomplicated falciparum malaria in pregnancy: methodological challenges
Source: Malar J. 2017 Dec 13;16:488. doi: 10.1186/s12936-017-2135-y (PMC5729448; doi:10.1186/s12936-017-2135-y)
Supplement: Supplementary file 2 — Additional file 2. PRISMA flowchart. [file 12936_2017_2135_MOESM2_ESM.pdf]

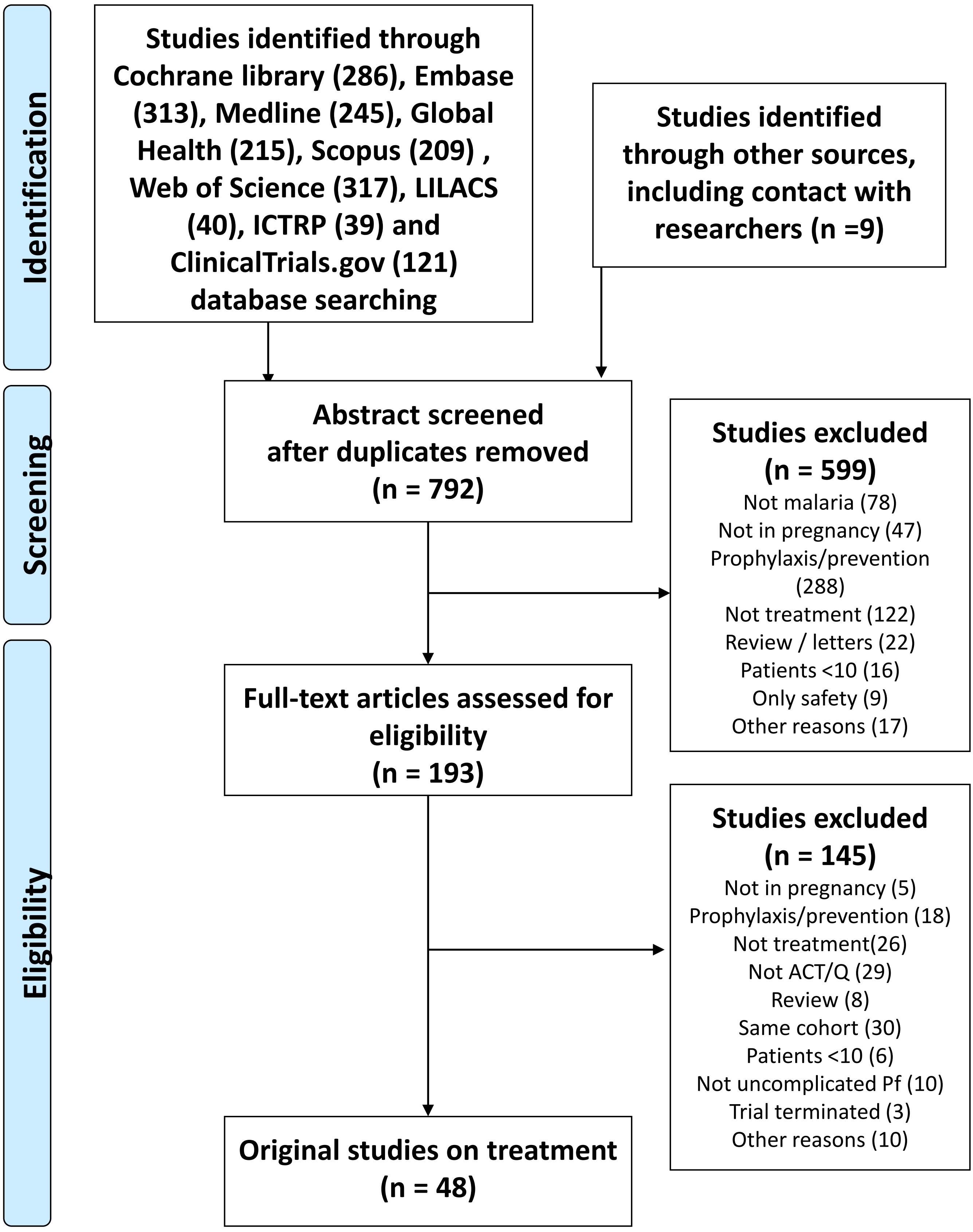

Additional file 2. The flowchart of the systematic literature search on treatment efficacy of uncomplicated falciparum malaria in pregnancy is shown according to the PRISMA statement.
