# Supplementary material for: Systematic literature review and meta-analysis of the efficacy of artemisinin-based and quinine-based treatments for uncomplicated falciparum malaria in pregnancy: methodological challenges
Source: Malar J. 2017 Dec 13;16:488. doi: 10.1186/s12936-017-2135-y (PMC5729448; doi:10.1186/s12936-017-2135-y)
Supplement: Supplementary file 3 — Additional file 3. Summary of the study design and reported outcomes. [file 12936_2017_2135_MOESM3_ESM.pdf]

Additional file 3. Summary of the study design and reported outcomes.

Study<br>Country<br>Study year	Study design<br>Treatment: N	Trimester	Efficacy endpoint reported	Intermittent preventive treatment	Dosing of treatment
Naing, 1988<br>Myanmar<br>1985-1986<br>[36]	RCT<br>Q: 23<br>AQ: 19	2,3	Fever clearance time<br>Parasite clearance time	Not applicable (not Sub-Saharan Africa)	Q: quinine dihydrochloride 10mg salt/kg x 3 times/day for 7 days orally or intravenously depending on the clinical condition
Harinasuta, 1990<br>Thailand<br>INA<br>[37] (conference abstract)	RCT<br>Q: 82<br>MFQ: 85	1,2,3	Parasite clearance on day 7	Not applicable (not Sub-Saharan Africa)	Q: quinine sulfate 600mg x 3times/day for 7 days
Nosten, 1993<br>Thailand<br>1989-1990<br>[23]	RCT<br>Q5: 16<br>Q5 + spiramycin: 16	2,3	Recurrence rate within 28 days<br>Parasite clearance time	Not applicable (not Sub-Saharan Africa)	Q: quinine sulphate 30mg salt/kg/day for 5 days<br>spiramycin: 1.2M IU x 3 times/day for 5 days
Sowunmi, 1998<br>Nigeria<br>1994-1997<br>[38]	RCT<br>AM: 23<br>AMMQ: 22	2,3	Cure rate on day 14 /28<br>Fever clearance time<br>Parasite clearance time<br>Peripheral parasitaemia of newborns at birth	Information not available	AM: 3.2 mg/kg given intramuscularly on day 0, followed by 1.6 mg/kg/day for 4 days<br>AMMQ: single dose of 3.2 mg/kg AM intramuscularly on day 0, followed by 7.5mg/kg/day MQ for 2 days
Bounyasong, 2001<br>Thailand<br>1995-1998<br>[39]	RCT<br>ASMQ: 29<br>Q: 28	2,3	Recrudescence within 28 days<br>Fever clearance time<br>Parasite clearance time	Not applicable (not Sub-Saharan Africa)	AS: Oral artesunate 2 mg/kg for the first dose, 1 mg/kg every 12 hours orally for 5 days minimally (The treatment might be extended until the parasites were absent and the patients had clinical improvement)<br>MQ: 25mg/kg (split on day 6: 15 mg/kg and 6 hours later 10 mg/kg)<br>Q: Quinine sulfate 10 mg/kg x 3 times/day for at least 7 days until the patients had clinically recovered

Additional file 3 continued.

Study<br>Country<br>Study year	Study design<br>Treatment: N	Trimester	Efficacy endpoint reported	Intermittent preventive treatment	Dosing of treatment
McGready, 2000<br>Thailand<br>1995-1997<br>[40]	RCT<br>Q: 42<br>ASMQ: 66	2,3	Cure rates on day 63<br>Fever clearance by 48h<br>Parasite clearance by 48h	Not applicable (not Sub-Saharan Africa)	Q: 10 mg salt/kg x 3 times/day for 7 days<br>AS (Guilin): 4mg/kg/day for 3 days<br>MQ (Lariam): 25 mg base/kg given as 15 mg/kg on day 1 and 10 mg/kg on day 2
McGready, 2001a<br>Thailand<br>1997-2000<br>[41]	RCT<br>QC: 65<br>AS: 64	2,3	Cumulative cure rate on day 42<br>Fever clearance by 48h<br>Parasite clearance by 48h<br>Gametocyte carriage	Not applicable (not Sub-Saharan Africa)	Q: 10 mg salt/kg x 3 times/day for 7 days<br>Clindamycin: 5 mg/kg x 3 times/day for 7 days<br>AS (Guilin): 2 mg/kg daily on days 0, 1, 2, 3 and 4 and 1 mg/kg daily on days 5 and 6 (total 10mg/kg)
McGready, 2005<br>Thailand<br>2001-2003<br>[42]	RCT<br>AAP: 39<br>Q: 42	2,3	Cumulative cure rate at delivery or on day 63<br>Fever clearance on day 2, 3 & 4<br>Parasite clearance time<br>Gametocyte carriage	Not applicable (not Sub-Saharan Africa)	AS (Guilin): 4mg/kg/day for 3 days (50mg tablet)<br>AP (Malarone): (atovaquone 20 mg/kg/day and 8 mg/kg/d) for 3 days (250+100mg tablet)<br>With 200mL of chocolate milk (8% fat)<br>Q: 10 mg salt/kg x 3 times/day for 7 days
Adam, 2004a<br>Sudan<br>2002-2003<br>[43]	RCT<br>Q (low dose): 18<br>Q (standard dose): 24	2,3	Failure rate within 28 days<br>Fever clearance time<br>Parasite clearance time	Information not available	Low dose group: quinine 10mg/kg x 2 times/day for 7 days<br>Standard dose group: quinine 10mg/kg x 3 times/day for 7 days<br>Quinine was administered intravenously until patients could tolerate
Kalilani, 2007<br>Malawi<br>2003-2004<br>[44]	RCT<br>ASSP: 47<br>SP: 47<br>SP + azithromycin: 47	2	Recrudescence rate within 28 days<br>Fever clearance on day 3<br>Parasite clearance on day 2 & 3<br>Parasitaemia at delivery<br>Prevalence of placental malaria	The second dose of the same drug was administered at least 4 weeks after the first dose instead of IPTp	AS: 200mg/day for 3 days<br>SP: 1500+75mg as a single dose
McGready, 2008<br>Thailand<br>2004-2006<br>[45]	RCT<br>AL: 125<br>AS: 128	2,3	Cumulative cure rate at delivery or on day 42<br>Fever clearance time<br>Parasite clearance time<br>Placental / cord blood malaria	Not applicable (not Sub-Saharan Africa)	AL (Coartem): (80+480mg) x 2 times/day for 3 days (at 0, 8, 24, 36, 48 and 60 h)<br>With 250mL of chocolate milk containing 7g of fat<br>AS (Guilin): 2mg/kg x 1 time/day for 7 days (50mg tablet)

Additional file 3 continued.

Study<br>Country<br>Study year	Study design<br>Treatment: N	Trimester	Efficacy endpoint reported	Intermittent preventive treatment	Dosing of treatment
Mutabingwa, 2009<br>Tanzania<br>2004-2006<br>[46]	RCT<br>ASAQ: 83<br>AQSP: 80<br>CD: 81<br>SP: 28	2,3	Recurrence rate within 28 days<br>Gametocyte carriage	Information not available	AQ: 10 mg/kg/day for 3 days<br>AS: 4 mg/kg/day for 3 days
Kaye, 2008<br>Uganda<br>2006<br>[47]	RCT<br>AL: 57<br>CD: 57	2,3	Cure rate on day 28<br>Fever clearance<br>Parasite clearance	Information not available	AL (Coartem): (80+400mg) x 2 times/day for 3 days<br>*Assumed to be 80+480mg
Piola, 2010<br>Uganda<br>2006-2009<br>[48]	RCT<br>AL: 152<br>Q: 152	2,3	Cure rate and cumulative recurrence by day 42<br>Cure rate and cumulative recurrence by delivery<br>Fever clearance on day 0, 1, 2 & 3<br>Parasite clearance on day 2 & 3<br>Gametocyte clearance within 7 days<br>Gametocyte carriage<br>Placental malaria	IPTp was interrupted while study period	AL: (80+480mg) x 2 times/day for 3 days<br>(at 0, 8, 24, 36, 48 and 60 h)<br>With 200mL milk<br>Q: quinine hydrochloride 10 mg base/kg x 3 times/day for 7 days (300 mg of the base per tablet)
Carmona-Fonseca, 2013<br>Colombia<br>2008-2011<br>[49]	RCT<br>AL: 15<br>ASMQ: 15	2,3	Cure rate on day 28	Not applicable (not Sub-Saharan Africa)	AL (80mg+480mg) x 2times/day for 3 days<br>AS 4 mg/kg x 1 time/day for 3 days (50mg tablet)<br>MQ 25 mg base/kg, divided into 2 or 3 days (250mg tablet)
D'Alessandro, 2016<br>Burkina Faso, Ghana, Malawi and Zambia<br>2010-2013<br>[50, 51]	RCT<br>AL: 880<br>ASAQ: 842<br>ASMQ: 848<br>DP: 853	2,3	Cure rate on day 63<br>Parasite clearance on day 1 & 2<br>Prevalence of placental malaria<br>Gametocytaemia	Some patients received IPTp-SP before the trial<br>IPTp-SP was restarted after 63 days	AL (Coartem): (80+480mg) x 2times/day for 3 days<br>With recommendation to take high-fat food or drinks together<br>ASAQ (Winthrop): (200+540mg) x 1 time/day for 3 days<br>ASMQ (Farmanguinhos): (200+440mg/day) x 1 time/day for 3 days<br>DP (Eurartesim): (120+960mg) x 1time/day for 3 days

Additional file 3 continued.

Study<br>Country<br>Study year	Study design<br>Treatment: N	Trimester	Efficacy endpoint reported	Intermittent preventive treatment	Dosing of treatment
Osarfo, 2017<br>Ghana<br>2011-2012<br>[52]	RCT<br>ASAQ: 205<br>DP: 212	2,3	Cure rate on day 42<br>Cure rate on day 28<br>Placental malaria	IPTp-SP was not given during the study period (42 days)	ASAQ (Arsuamoon)<br>AS 4mg/kg and AQ 10mg/kg in twelve hourly doses over 3 days<br>DP (P-alaxin)<br>Total dosing of 6.75mg/kg DHA and 55mg/kg PPQ over 3 days rounded to the nearest half tablet
Onyamboko, 2015<br>DRC<br>2013-2014<br>[53] (conference abstract)	RCT<br>AL: 48<br>AL5: 48	2,3	Cure rate on day 42<br>Placental malaria	Information not available	AL for 3 days (6 doses)<br>AL for 5 days (10 doses)<br>With milk
Ukah, 2015<br>Nigeria<br>2013<br>[54]	RCT<br>AL: 75<br>ASAQ: 75	2,3	Cure rate on day 28<br>Symptom clearance time<br>Parasite clearance by day 3	IPTp-SP x 2	AL (GloatemForte): (80+480mg) x 2 times/day for 3 days<br>ASAQ (Winthrop): (100+270mg) x 2 times/day for 3 days
Iribhogbe, 2017a<br>Nigeria<br>2013-2014<br>[55]	RCT<br>AL: 40<br>ASAQ: 40	2,3	Recurrence within 28 days<br>Fever clearance on day 3<br>Parasite clearance on day 3	Information not available	AL: (80+480mg) x 2 times/day for 3 days<br>ASAQ: (300/810mg) x once a day for 3 days
CTRI/2009/091/001055<br>India<br>2010-2015<br>[56] (registered trial)	RCT<br>ASMQ (500)<br>ASSP (500)<br>* Planned number	2,3	Cure rate on day 63<br>Recurrence by day 63	Not applicable (not Sub-Saharan Africa)	AS: 200mg/day for 3 days<br>MQ: 440mg/day for 3 days<br>AS: 200mg/day for 3 days<br>SP: 1500+75mg single dose on day1

Additional file 3 continued.

Study<br>Country<br>Study year	Study design<br>Treatment: N	Trimester	Efficacy endpoint reported	Intermittent preventive treatment	Dosing of treatment
NCT01054248<br>Thailand<br>2010-2016<br>[57] (registered trial)	RCT<br>AL4 (335)<br>ASMQ (335)<br>DP (335)<br>*Planned number of participants including vivax malaria patients	2,3	Cure rate on day 63 or until delivery<br>Gametocyte carriage<br>Placental malaria	Not applicable (not Sub-Saharan Africa)	AL(Coartem): (100+600mg) x 2 times/day for 4 days (at 0, 8, 24, 36, 48, 60, 72 and 84 hours) with 100mL chocolate milk<br>AS(Guillin Factory no.1, 50mg/tablet): 4mg/kg/day for 3 days (rounded to the nearest quarter tablet)<br>MQ(Mequin, 250mg/tablet): 8mg/kg/day for 3 days (rounded to the nearest quarter tablet)<br>ASMQ(Farmanguinhos: 100+220mg/tablet): 2 tablets/day for 3 days (if this fixed combination is available)<br>DP (Holley-Cotec:40+320mmg/tablet): (2.4 mg/kg DHA and 20 mg/kg PPQ) x 1 time/day for 3 days (rounded to the nearest half tablet)
McGready, 2003a<br>Thailand<br>2000-2001<br>[58]	PK<br>AAP: 24	2,3	Cure rate on day 28<br>Fever clearance by 48h<br>Parasite clearance time	Not applicable (not Sub-Saharan Africa)	AS (Guilin): 4mg/kg/day for 3 days (50mg tablet)<br>AP (Malarone): (atovaquone 20 mg/kg/day and 8 mg/kg/d) for 3 days (250+100mg tablet)<br>With 200mL of chocolate milk (8% fat)
Adam, 2012<br>Sudan<br>2007-2008<br>[59]	PK study<br>DP: 12<br>(control: 12 matched non-pregnant women)	2,3	Cure rate on day 63<br>Fever clearance within 2 days<br>Parasite clearance within 2 days	Information not available	DHA/PQ-phosphate (DuoCotecxin): 2.4 mg of DHA/kg/day and 20 mg of PQ (as phosphate)/kg/day rounded to the nearest half tablet for 3 days
Onyamboko, 2011<br>DRC<br>2007-2008<br>[60]	PK<br>ASSP: 24<br>(control: 14 non-pregnant women)	2,3	Recurrence within 28 days<br>Parasite clearance by 12 h	Information not available	AS (Guilin): 200mg once<br>SP: 1725mg 24 hours after AS administration

Additional file 3 continued.

Study<br>Country<br>Study year	Study design<br>Treatment: N	Trimester	Efficacy endpoint reported	Intermittent preventive treatment	Dosing of treatment
McGready, 2012<br>Thailand<br>2008-2009<br>[61]	PK<br>AS: 20	2,3	Cumulative cure rate on day 63<br>Fever clearance time<br>Parasite clearance time	Not applicable (not Sub-Saharan Africa)	Group 1: intravenous AS (Guilin) 4mg/kg on day 0 followed by oral AS 4mg/kg for 6 days<br>Group 2: oral AS (Guilin) 4mg/kg on day 0 followed by intravenous AS 4mg/kg on day 1 followed by oral artesunate 4mg/kg daily for 5 days
Rijken, 2011<br>Thailand<br>2008<br>[62]	PK<br>DP: 24<br>(control: 24 matched non-pregnant women)	2,3	Cumulative cure rate on day 63<br>Fever clearance<br>Parasite clearance<br>Placental malaria	Not applicable (not Sub-Saharan Africa)	DP (Holley Pharm)<br>Total dosing of 6.5 mg/kg DHA and 51.2mg/kg PPQ over 3 days<br>The tablets (40+320mg) were divided to the nearest quarter
Valea, 2014<br>Burkina Faso<br>2008-2009<br>[63]	PK<br>ASMQ: 24<br>(control: 24 non-pregnant women)	2,3	Cure rate on day 63<br>Parasite clearance on day 3	IPTp-SP x 2	ASMQ (Farmanguinhos)<br>AS: 3.6mg/kg x 1 time/day for 3 days<br>MQ: 8mg/kg x 1 time/day for 3 days<br>(<50kg: 150+330mg/day<br>50-60kg: 200+440mg/day<br>>60kg: 250+550mg/day)
Juma, 2014<br>Kenia<br>2012<br>[64] (conference abstract)	PK<br>AL: 45<br>(control: 25 non-pregnant women)	2,3	Treatment response on day 28	Information not available	AL: (80+480mg) x 2 times/day for 3 days<br>(at hours 0, 8, 24, 36, 48 and 60)
Mosha, 2014<br>Tanzania<br>2012<br>[65]	PK<br>AL: 33	2,3	Cure rate / recurrence rate on day 42<br>Number of recrudescence	Information not available	AL (Coartem): (80+480mg) x 2 times/day for 3 days<br>(at 0, 8, 24, 36, 48 and 60 h)<br>With 200mL of milk containing 4.5g of fat
Nyunt, 2016<br>Uganda<br>2013-2014<br>[66]	PK<br>AL: 30<br>(control: 30 non pregnant adults)	2,3	Cure rate / recurrence rate on day 42<br>Parasite clearance on day 2	SP x 2 (16-24w, 28-36 weeks)	AL (Coartem): (80+480mg) x 2 times/day for 3 days<br>With 200mL milk

Additional file 3 continued.

Study<br>Country<br>Study year	Study design<br>Treatment: N	Trimester	Efficacy endpoint reported	Intermittent preventive treatment	Dosing of treatment
Mutagonda, 2017<br>Tanzania<br>2014-2015<br>[67, 68]	PK<br>AL: 92	2,3	Cure rate / recurrence rate on day 28<br>(Cumulative cure rate on day 28 was reported in sub-cohort [66])	Information not available	AL (Coartem): (80+480mg) x 2 times/day for 3 days<br>(at 0, 8, 24, 36, 48 and 60 h)<br>With milk containing 4.5g of fat
Adam, 2004b<br>Sudan<br>2000-2002<br>[69]	Single-arm<br>Q: 26	1	Cure rate on day 28<br>Fever clearance within 3 days<br>Parasite clearance within 3 days	Information not available	Q: 30 mg salt/kg/day for 7 days<br>Quinine was administered intravenously until patients could tolerate
Adam, 2004c<br>Sudan<br>1997-2001<br>[70]	Single-arm<br>AM im: 28	1,2,3	Number of recurrence<br>Parasite clearance on day 7	Information not available	AM(Kunming): given intramuscularly, 80mg initially then 80mg after 12 h, then 80mg daily (total 480 mg)
Adegnika, 2005<br>Gabon<br>2003-2004<br>[71]	Single-arm<br>Q: 50	2,3	Cure rate on day 28<br>Cure rate on day 56<br>Prevalence of placental malaria	Information not available	Q:10 mg salt/kg x 3 times/day for 7 days rounded to the nearest half tablet of 300mg quinine
Adam, 2006<br>Sudan<br>2004-2005<br>[72]	Single-arm<br>ASSP: 32	2,3	Cure rate on day 28<br>Fever clearance within 2 days<br>Parasite clearance within 2 days	Information not available	AS: 200mg/day for 3 days on days 0—2<br>SP: 1500+75mg given as a single dose on day 0
Ndiaye, 2011<br>Senegal<br>2009-2011<br>[73] (conference abstract)	Single-arm<br>ASAQ: 28	2,3	Recurrence rate within 42 days<br>Parasite clearance<br>Fever clearance<br>Placental malaria<br>Congenital malaria	Information not available	ASAQ (Coarsucam)<br>AS: 200mg/day for 3 days<br>AQ: 540mg/day for 3 days

Additional file 3 continued.

Study<br>Country<br>Study year	Study design<br>Treatment: N	Trimester	Efficacy endpoint reported	Intermittent preventive treatment	Dosing of treatment
Iribhogbe, 2017b<br>Nigeria<br>2013-2014<br>[74]	Single-arm<br>ASAQ: 50	2,3	Recurrence within 28 days	INA	AS: 4mg/kg/day for 3 days<br>AQ: 10mg/kg/day for 3 days
McGready, 1998a<br>Thailand<br>1992-1996<br>[75]	Observational cohort<br>AS: 61<br>AMMQ: 3<br>ASMQ: 26	1,2,3	Recurrence within 28 days<br>Parasite clearance on day 7<br>Gametocyte carriage	Not applicable (not Sub-Saharan Africa)	AS (Guilin): 4 mg/kg on day 0, 2 mg/kg on days 1 and 2, and 1 mg/kg on days 3-6 (total dose of 12 mg/kg) or 4 mg/kg/day for 3 days<br>Mefloquine (Laham): 25 mg/kg, usually as a split regimen on days 5 and 6, was added when there had been no previous intake of mefloquine during the pregnancy<br>AM (Kunming): a total dose of 12 mg/kg over 3 or 5 d. AM was given intramuscularly if the patient could not tolerate oral treatment
McGready, 1998b<br>Thailand<br>1991-1996<br>[76]	Observational cohort<br>Q<br>Primary treatment: 113<br>Retreatment: 91	1,2,3	Recurrence within 42 days<br>Parasite clearance on day 3<br>Fever clearance at 48h<br>Gametocyte carriage	Not applicable (not Sub-Saharan Africa)	Q: 30 mg salt/kg/day for 7 days
McGready, 2001b<br>Thailand<br>1992-2000<br>[77]	Observational cohort artemisinins<br>Primary treatment: 229<br>Retreatment: 310	1,2,3	Cumulative recurrence rate within 42 days<br>Proportion of recurrence within 42 days<br>Fever clearance time<br>Parasite clearance time<br>Gametocyte carriage	Not applicable (not Sub-Saharan Africa)	AS (Guilin): 12-14mg/kg in total over 3 or 7 days<br>MQ (Lariam): 25 mg base/kg given over 2 days<br>AP (Malarone): (atovaquone 20 mg/kg/day and 8 mg/kg/d) for 3 days

Additional file 3 continued.

Study<br>Country<br>Study year	Study design<br>Treatment: N	Trimester	Efficacy endpoint reported	Intermittent preventive treatment	Dosing of treatment
Laochan, 2015<br>Thailand<br>1994-2009<br>[78]	collation of an observational cohort and interventional studies‡<br>AS: 151, AAP: 11, AC: 143, AL: 43, ASMQ: 5, DP 29, Q: 476, QC: 29 MQ: 22	1,2,3	Description of recurrent episodes of malaria with PCR	Not applicable (not Sub-Saharan Africa)	Q: 10 mg salt/kg x 3 times/day for 7 days<br>AS (Guilin): 2 mg/kg x 1 time/day for 7 days (total dose 10–16 mg/kg)<br>AS (Guilin): 4mg/kg x 1 time/day for 3 days<br>AL (Coartem): (80+480mg) x 2 times/day for 3 days<br>MQ: 25 mg/kg stat dose or over two days<br>DP (Duo-Cotexcin): DHA 3 mg/kg, PPQ 15–17 mg/kg x 1 time/day for 3 days<br>AP (Malarone): (atovaquone 20 mg/kg/day and 8 mg/kg/d) for 3 days<br>Clindamycin: 300mg x 3 times/day for 7 days
McGready, 2002<br>Thailand<br>1995-2000<br>[79]	observational<br>Q<br>novel: 25<br>retreatment: 209	1	Recurrence within 28 days<br>Fever clearance<br>Parasite clearance<br>Gametocyte carriage	Not applicable (not Sub-Saharan Africa)	Q: 30 mg salt/kg/day for 7 days
McGready, 2003b<br>Thailand<br>1999-2001<br>[80]	Observational cohort<br>AAP: 27	1,2,3	Recrudescence within day 42<br>Fever clearance by 48h<br>Parasite clearance time<br>Gametocyte carriage<br>Placental malaria	Not applicable (not Sub-Saharan Africa)	AS: 4mg/kg/day for 3 days or 2mg/kg/day for 7 days (with a loading dose of 4 mg/kg in hyperparasitaemic patients with >4%)<br>AP: (atovaquone 20 mg/kg/day and 8 mg/kg/d) for 3 days
Villegas, 2005<br>Venezuela<br>2002-2005<br>[81] (conference abstract)	Observational cohort<br>ASMQ: 27	INA	INA (efficacy)	Not applicable (not Sub-Saharan Africa)	ASMQ for 2-3 days
Rijken, 2008<br>Thailand<br>2006-2007<br>[82]	Observational cohort<br>DP: 62	1,2,3	Cumulative cure rate by day 63<br>Fever clearance on day 2<br>Parasite clearance time	Not applicable (not Sub-Saharan Africa)	DP (Holley Pharm)<br>Total dosing of 6.5 mg/kg DHA and 51.2mg/kg PPQ over 3 days (40+320mg tablet)

Additional file 3 continued.

Study<br>Country<br>Study year	Study design<br>Treatment: N	Trimester	Efficacy endpoint reported	Intermittent preventive treatment	Dosing of treatment
Rulisa, 2012<br>Rwanda<br>2007-2009<br>[83]	Observational cohort<br>AL: (1072)†	1,2,3	Cure rate on day 56<br>Recurrence within 56 days	23.4% received IPTp-SP	Rwanda National Guideline
Kalilani, 2013 and Cohee, 2016<br>Malawi<br>2009-2010<br>[84, 85]	Observational cohort<br>AL: 56	1,2	Recrudescence rate by delivery<br>Placental malaria<br>Temporal relationship between peripheral parasitaemia and placental malaria	IPTp-SP x 3	AL: (80+480mg) x 2 times/day for 3 days<br>(at 0h, 8h, day2 AM, day2 PM, day3 AM, day3 PM)

AAP: artesunate-atovaquone-proguanil, AC: artesunate-clindamycin, AL: artemether-lumefantrine, AQ: amodiaquine, AS: artesunate, CD: chlorproguanil-Dapsone, CHQ: chloroquine, DP: dihydroartemisinin(DHA)-piperaquine(PPQ), DRC: Democratic Republic of Congo, ECG: electrocardiography, INA: information not available, MQ: mefloquine, N: number of pregnant women included, PCR: polymerase chain reaction, PK: pharmacokinetic study, Q: quinine, QC: quinine-clindamycin, RCT: randomised controlled trial, SP: sulfadoxine-pyrimethamine. *Planned number of participants in clinical trial registry. NCT01054248 includes P. vivax patients. † This number includes patients without parasitological confirmation. The exact number of parasitologically confirmed patients is not known. ‡ This study included patients enrolled in other studies [40-42, 45, 58, 61, 61, 80, 82].
