# Supplementary material for: Systematic literature review and meta-analysis of the efficacy of artemisinin-based and quinine-based treatments for uncomplicated falciparum malaria in pregnancy: methodological challenges
Source: Malar J. 2017 Dec 13;16:488. doi: 10.1186/s12936-017-2135-y (PMC5729448; doi:10.1186/s12936-017-2135-y)
Supplement: Supplementary file 4 — Additional file 4. Summary of the quality of studies. [file 12936_2017_2135_MOESM4_ESM.pdf]

Additional file 4. Summary of the quality of studies

Study [reference]	Study design	Randomisation	Allocation concealment	Blinding	Loss to follow-up for efficacy assessment
Naing, 1988 [36]	RCT	Unclear: 'Patients were randomised […] depending on clinical severity and parasite density.'	Unclear	Unclear	Unclear
Harinasuta, 1990 [37]*	RCT	INA	INA	INA	0/82 (Q)<br>0 85 (MQ)
Nosten, 1993 [23]	RCT	Yes, but the method is not specified.	Unclear	At least patients were blinded by using identical placebo.	11/43 were dropped out.
Sowunmi, 1998 [38]	RCT	Yes, but the method is not specified.	Unclear	Unclear	Unclear
Bounyasong, 2001 [39]	RCT	Yes, but the method is not specified.	Unclear	Unclear	1/30 (Q)<br>2/30 (ASSP)
McGready, 2000 [40]	RCT	Block randomisation	Unclear	Open-label	0/66 (ASMQ)<br>0/42 (Q)
McGready, 2001a [41]	RCT	Block randomisation	Unclear	Open-label	19/65 (QC)<br>17/64 (AS)
McGready, 2005 [42]	RCT	Computer-generated block randomisation	Sealed envelope	Open-label	0/39 (AAP)<br>0/42 (Q)
Adam, 2004a [43]	RCT	Yes, but the method is not specified.	Unclear	Unclear	7/25 (20mg/kg/day)<br>2/26 (30mg/kg/day)
Kalilani, 2007 [44]	RCT	Block randomisation	Sealed envelope	Open-label<br>Lab technicians were blinded.	8/47 (ASSP)<br>7/47 (SP)<br>5/47 (SP + azithromycin)
McGready, 2008 [45]	RCT	Block randomisation	Sealed envelope	Open-label<br>Lab technicians and patient examiners were blinded.	5/125 (AL)<br>3/125 (AS)
Mutabingwa, 2009 [46]	RCT	Block randomisation	Sealed envelope	Open-label<br>Microscopists were blinded.	8/83 (ASAQ)<br>8/80 (AQSP)<br>4/81 (CD)<br>2/28 (SP)
Kaye, 2008 [47]	RCT	Computer-generated random number	Sealed envelope	Open-label	5/57 (AL)<br>5/57 (CD)
Piola, 2010 [48]	RCT	Computer-generated permuted block of eight	Sealed envelope	Open-label<br>Microscopists and clinical investigators were blinded.	10/152 (Q)<br>6/152 (AL)
Carmona-Fonseca, 2013 [49]	RCT	Ballot system	Yes	Unclear	0/15 (AL)<br>0/15 (ASMQ)
D'Alessandro, 2016 [50, 51]	RCT	Block randomisation	Sealed envelope	Open-label<br>Lab technicians (interpretation of PCR) were blinded.	50/880 (AL)<br>51/842 (ASAQ)<br>94/853 (DP)<br>77/848 (ASMQ)
Osarfo, 2017 [52]	RCT	Computer-generated random number	Sealed envelope	Open-label.<br>Microscopists were blinded.	24/206 (ASAQ)<br>18/212 (DP)
Onyamboko, 2015 [53]*	RCT	Yes, but the method is not specified.	INA	Open-label	INA
Ukah, 2015 [54]	RCT	Computer-generated	Only pharmacy had access to the allocation sequence.	Double blind	3/75 (AL)<br>7/75 (ASAQ)

Additional file 4 continued.

Study [reference]	Study design	Randomisation	Allocation concealment	Blinding	Loss to follow-up for efficacy assessment
Iribhogbe, 2017a [55]	RCT	Balloting	Unclear	Open-label	2/40 (AL)<br>3/40 (ASAQ)
CTRI/2009/091/001055 [56]*	RCT	Yes, but the method is not specified.	INA	INA	INA
NCT01054248 [57]*	RCT	Computer generated permuted block of 15	sealed envelope	Open-label Microscopists were blinded.	INA
McGready, 2003a [58]	PK study	Not applicable	Not applicable	Unclear	8/24
Adam, 2012 [59]	PK study	Not applicable	Not applicable	Unclear	0/12
Onyamboko, 2011 [60]	PK study	Not applicable	Not applicable	Unclear	0/26
McGready, 2012 [61]	PK study	No: assigned alternatively	Not applicable	Unclear	0/20
Rijken, 2011 [62]	PK study	Not applicable	Not applicable	Unclear	2/24 (delivery)
Valea, 2014 [63]	PK study	Not applicable	Not applicable	Unclear	1/25
Juma, 2014 [64]	PK study	Not applicable	Not applicable	Open-label	INA
Mosha, 2014 [65]	PK study	Not applicable	Not applicable	Unclear	0/33
Nyunt, 2016 [66]	PK study	Not applicable	Not applicable	No	
Mutagonda, 2017 [67, 68]	PK study	Not applicable	Not applicable	Unclear	10/92
Adam, 2004b [69]	Single-arm	Not applicable	Not applicable	Unclear	0/26
Adam, 2004c [70]	Single-arm	Not applicable	Not applicable	Unclear	0/28
Adegnika, 2005 [71]	Single-arm	Not applicable	Not applicable	Unclear	0/50 (day 28)<br>9/50 (day 56)
Adam, 2006 [72]	Single-arm	Not applicable	Not applicable	Unclear	0/36
Ndiaye, 2011 [73]*	Single-arm	Not applicable	Not applicable	Open-label	0/28
Iribhogbe, 2017b [74]	Single-arm	Not applicable	Not applicable	Open-label	8/50
McGready, 1998a [75]	Observational cohort	Not applicable	Not applicable	Unclear	5/83 (all)
McGready, 1998b [76]	Observational cohort	Not applicable	Not applicable	Unclear	Unclear
McGready, 2001b [77]	Observational cohort	Not applicable	Not applicable	Unclear	Unclear
Laochan, 2015 [78]	Collation of observational cohorts and RCTs [40-42, 45, 58, 61, 61, 80, 82	refer to each study	refer to each study	refer to each study	refer to each study
McGready, 2002 [79]	Observational cohort	Not applicable	Not applicable	Unclear	Unclear
McGready, 2003b [80]	Observational cohort	Not applicable	Not applicable	Unclear	6/27
Villegas, 2005 [81]*	Observational cohort	Not applicable	Not applicable	INA	INA
Rijken, 2008 [82]	Observational cohort	Not applicable	Not applicable	Unclear	5/50 (delivery)
Rulisa, 2012 [83]	Observational cohort	Not applicable	Not applicable	Unclear	Unclear
Kalilani, 2013 [84, 85]	Observational cohort	Not applicable	Not applicable	Unclear	INA

AAP: artesunate-atovaquone-proguanil, AL: artemether-lumefantrine, AQ: amodiaquine, AS: artesunate, CD: chlorproguanil-Dapsone, DP: dihydroartemisinin-piperaquine, INA: information not available (conference abstract or registered trial). MQ: mefloquine, PK: pharmacokinetic, Q: quinine, QC: quinine-clindamycin, RCT: randomised controlled trial, SP: sulfadoxine-pyrimethamine. * conference abstract or registered trial.
