# Supplementary material for: Systematic literature review and meta-analysis of the efficacy of artemisinin-based and quinine-based treatments for uncomplicated falciparum malaria in pregnancy: methodological challenges
Source: Malar J. 2017 Dec 13;16:488. doi: 10.1186/s12936-017-2135-y (PMC5729448; doi:10.1186/s12936-017-2135-y)
Supplement: Supplementary file 5 — Additional file 5. Summary of treatment administration and interruption. [file 12936_2017_2135_MOESM5_ESM.pdf]

Additional file 5. Summary of treatment administration and interruption

The treatment administration was supervised fully in 31 studies and partially in 5 studies [47,52,67,71,79] (Table 2). In one study the treatment administration was not supervised [83] and it was not clearly reported in eleven studies [37,49,54-56,66,73,74,76,78,84]. In 16 studies with AL, seven studies co-administered fat (i.e. milk) to increase the bioavailability [19,45,48,53,57,66,67] and one study recommended patients to take high fat food [50]. The other eight studies did not specify the concurrent fat intake.

Treatment interruption before the completion of the treatment due to adverse reaction or withdrawal of consent was reported in six out of 31 studies with full supervision (19%, 6/31). Two RCTs reported the proportion of patients whose treatment was interrupted during ABT and QBT. The proportion of treatment interruption was 0% (0/64) with AS for seven days and 3% (2/67) with QC for seven days;[41] and 0% (0/152) with AL for three days and 3% (4/152) with Q for seven days [48]. Treatment disruption during QBT for seven days was 18% (9/51) in another study [43]. Treatment disruption during ABT was reported in three other studies: 0.8% (1/128 with AS for seven days) [45], 0% (0/125 with AL) [45], 3% (1/35 with AL) [65], and 3% (1/30 with ASAQ) [73].

To compare the acute adverse events of ABT and QBT, the results from five RCTs directly comparing them were reviewed. Meta-analysis of five RCTs comparing ABT versus QBT revealed that hypoglycemia, tinnitus, nausea and vomitting were more frequently reported in QBT than in ABT (Supplementary 12). Tinnitus was reported in 35–80% of the women treated with QBT [39-43,48,76,79] and in 0–30% of the women treated with ABT [40-42,45,48,52,74,77,80] except one study reporting 64% for ASMQ [39].
