# Supplementary material for: Systematic literature review and meta-analysis of the efficacy of artemisinin-based and quinine-based treatments for uncomplicated falciparum malaria in pregnancy: methodological challenges
Source: Malar J. 2017 Dec 13;16:488. doi: 10.1186/s12936-017-2135-y (PMC5729448; doi:10.1186/s12936-017-2135-y)
Supplement: Supplementary file 6 — Additional file 6. The number of study arms with PCR-corrected outcomes for each comparison of drugs. [file 12936_2017_2135_MOESM6_ESM.pdf]

	Q	QC	AS	AM	AL	ASAQ	ASMQ	DP	AAP	AC	ASSP	AMMQ
Q	2 (8)				1		2		1			
QC			1									
AS			1 (1)		1							
AM				1								(1)
AL					7 (1)	1 (2)	2 [1]	1 [1]				
ASAQ						2 (1)	1	1 (1)				
ASMQ							1 (2)	1 [1]			[1]	
DP								3				
AAP									2			
AC												
ASSP											2 (1)	
AMMQ												(1)

Additional file 6. The number of study arms with PCR-corrected outcomes for each comparison of drugs. The number of studies in which PCR-corrected outcomes were not available is shown in round brackets. The number of unpublished registered trials is shown in square brackets. The numbers of non-comparative studies were shown on the diagonal. Two observational studies which include more than five treatment groups (McGready, 2001b [77] and Laochan, 2015 [78]) were excluded from this figure. AAP: artesunate + atovaquone-proguanil, AC: artesunate + clindamycin, AL: artemether-lumefantrine, AM: artemether, AS: artesunate, AQ: amodiaquine, MQ: mefloquine, SP: sulfadoxine-pyrimethamine, DP: dihydroartemisinin-piperaquine, Q: quinine, QC: quinine + clindamycin.
