# Supplementary material for: Systematic literature review and meta-analysis of the efficacy of artemisinin-based and quinine-based treatments for uncomplicated falciparum malaria in pregnancy: methodological challenges
Source: Malar J. 2017 Dec 13;16:488. doi: 10.1186/s12936-017-2135-y (PMC5729448; doi:10.1186/s12936-017-2135-y)
Supplement: Supplementary file 7 — Additional file 7. Background information of patients at inclusion. [file 12936_2017_2135_MOESM7_ESM.pdf]

Additional file 7. Background information of patients at inclusion.

Study [reference]	Treatment group	N	Age (year)	Weight (kg)	Gestational age at malaria (week)	Gravidity	Parity	Parasitaemia (/μL)	Haemoglobin (g/dL)	Body temperature (°C)	Note
Naing, 1988 [36]	Q	23	26.7 ± 1.2	NA	2nd tri (50%)<br>3rd tri (50%)	NA	0 (41%)<br>1–3 (31%)<br>≥4 (29%)	Log mean 4.3 ± 0.3	NA	NA	Gestational age and parity includes patients treated with amodiaquine.
Harinasuta, 1990 [37]	Q	82	NA	NA	1st tri (9%)<br>2nd tri (59%)<br>3rd tri (33%)	NA	NA	NA	NA	NA	Conference abstract
Nosten, 1993 [23]	Q5	16	28 ± 6.8	48 ± 5.1	19.3 ± 6.5	NA	4*	631‡<br>(91–8256)	Hct 26 ± 4 %	NA	
	Q5+spiramycin	16	26 ± 6.2	49 ± 5.5	21.7 ± 9		2*	537‡<br>(53–5512)	Hct 28 ± 5 %		
Sowunmi, 1998 [38]	AM	23	29.9 ± 6.1<br>(21–41)	63.9 ± 12.1<br>(50–119)	NA	NA	0 (65%)<br>1 (13%)<br>2 (9%)<br>≥3 (13%)	29719‡<br>(1674–250138)	Anaemia[a] 30%	38.1 ± 0.2<br>(37.5–39.0)	
	AM-MQ	22	28.9 ± 5.1<br>(20–40)	64.1 ± 11.9<br>(53–121)			0 (64%)<br>1 (18%)<br>2 (5%)<br>≥3 (14%)	28044‡<br>(2001–199992)	Anaemia[a] 30%	38.1 ± 0.1<br>(36.7–39.3)	
Bounyasong, 2001 [39]	Q	29	27.2	58.7	27.4	NA	1.6	1313.35 /200WBC	Hct 35.2 %	NA	Body weight before pregnancy.
	ASMQ	28	26.1	57.1	26.7		1.4	1329.68 /200WBC	Hct 34.3 %		
McGready, 2000 [40]	ASMQ	66	24* (15–37)	48* (36–68)	24* (12–40)	NA	1 (27%)<br>1 (29%)	11651‡<br>(32–241127)	Hct 29.4*<br>(17.5–39.0) %	Fever 15%	
	Q	42	23* (16–36)	50* (41–67)	24* (15–38)			19086‡<br>(79–149389)	Hct 28.7*<br>(21.0–37.2) %	Fever 21%	
McGready, 2001a [41]	QC	65	25* (15–41)	49*<br>(40–64)	26* (16–40)<br>28* (16–40)	NA	1 (26%)<br>1 (27%)	11099‡<br>(16–93019)	Hct 29.2*<br>(14.9–38.5) %	Fever 22%	
	AS	64	24* (15–41)	49*<br>(39–74)				9822‡<br>(16–109724)	Hct 29.6*<br>(20.0–38.9) %	Fever 14%	
McGready, 2005 [42]	Q	42	26 ± 7<br>(16–39)	50 ± 7<br>(30–68)	21 ± 4.5<br>(14.9–30.2)	1 (26%)	NA	2084‡<br>(33–109648)	Anaemia[a]<br>45.2%	Fever 74%	History of fever.
	AAP	39	26 ± 6<br>(16–37)	49 ± 7<br>(40–69)	21 ± 5.3<br>(10.1–36.2)	1 (31%)		2596‡<br>(33–123027)	Anaemia[a]<br>38.5%	Fever 59%	
Adam, 2004a [43]	Q 20mg/kg/d	18	25.2 ± 6.0	59.3 ± 15.9	26.1 ± 9.9	NA	2.6 ± 1.9	5837 ± 8361	8.9 ± 0.6	38.1 ± 0.9	
	Q 30mg/kg/d	24	25.5 ± 1.9	52.2 ± 9.3	26.1 ± 8.9		2.6 ± 2.2	4207 ± 12325	8.6 ± 0.9	37.8 ± 1.0	

Additional file 7 continued.

Study [reference]	Treatment group	N	Age (year)	Weight (kg)	Gestational age at malaria (week)	Gravidity	Parity	Parasitaemia (/µL)	Haemoglobin (g/dL)	Body temperature (°C)	Note
Kalilani, 2007 [44]	ASSP	47	20* [17–24]	52.9 ± 5.9	22* [20–26]	1 (60%)<br>2 (9%)<br>≥3 (32%)	NA	686.6‡<br>(120–4260)	10.5 ± 1.6	Fever 4%	Summary of all 47 enrolled patients. 7 HIV-positive cases.
McGready, 2008 [45]	AL	125	26 ± 7<br>(14–42)	50 ± 6<br>(35–65)	23.7 ± 6.8<br>(13.1–39.2)	3* (1–13)	1* (0–9)	3548‡<br>(65–457088)	Hct<br>30.0 ± 4.4 %	36.8 ± 1.0	
	AS	128	27 ± 8<br>(14–44)	49 ± 7<br>(3?–71)	24.8 ± 7.6<br>(13.1–39.4)	3* (1–11)	2* (0–9)	3162‡<br>(48–158489)	Hct<br>29.9 ± 5.0 %	37.0 ± 1.1	
Mutabingwa, 2009 [46]	AL	83	21* [19–26]	NA	6* [5–7] months	NA	1 (59%)	181* [62–628] /200WBC	9.3 ± 1.3	NA	All HIV negative.
Kaye, 2008 [47]	AL	57	23.5 ± 5.29	NA	2nd tri (25.5%)<br>3rd tri(74.5%)	1 (33%)<br>2 (26%)<br>≥3 (42%)	NA	24900 ± 3368.2	NA	36.9	Summary of all 57 enrolled patients.
Piola, 2010 [48]	Q<br>AL	152<br>152	22.6 (17–38)<br>22.5 (15–38)	58 (IQR 10)<br>58 (IQR 10)	22.3 (9–38)<br>24.7 (10–39)	2* (1–7)<br>2* (1–8)	NA	1995‡ (IQR 9771)<br>1418‡ (IQR 4727)	10.9*<br>10.9*	Fever 20%<br>Fever 23%	
Carmona-Fonseca, 2013 [49]	AL & ASMQ	30	22.0 ± 6.7	NA	2nd tri (50%)<br>3rd tri (50%)	NA	1 (42%)<br>2 (18%)<br>≥3 (40%)	NA	10.6 ± 1.2	NA	Age, parity and haemoglobin are the average of those of 60 P.vivax cases and 30 P.f cases.
D'Alessandro, 2016 [50]	AL	880	22.6 ± 5.6	NA	2nd tri (71.8%)<br>3rd tri (28.2%)	1 (36.3%)<br>2 (23.1%)<br>≥3 (40.6%)	NA	800*<br>[213–2880]	10.2*<br>[9.2–11.0]	Fever 6.5%	Patients taking antiretroviral treatment were not enrolled.
	ASAQ	842	23.4 ± 5.9		2nd tri (75.0%)<br>3rd tri (24.9%)	1 (37.4%)<br>2 (22.2%)<br>≥3 (40.4%)		569*<br>[165–2025]	10.1*<br>[9.1–11.0]	Fever 6.8%	
	DP	853	22.3 ± 5.4		2nd tri (68.5%)<br>3rd tri (31.5%)	1 (40.0%)<br>2 (25.3%)<br>≥3 (34.7%)		680*<br>[200–2760]	10.1*<br>[9.1–11.0]	Fever 3.2%	
	ASMQ	848	23.5 ± 5.9		2nd tri (65.8%)<br>3rd tri (34.2%)	1 (32.7%)<br>2 (23.7%)<br>≥3 (43.6%)		840*<br>[218–3040]	10.1*<br>[9.1–10.9]	Fever 8.0%	

Additional file 7 continued.

Study [reference]	Treatment group	N	Age (year)	Weight (kg)	Gestational age at malaria (week)	Gravidity	Parity	Parasitaemia (/µL)	Haemoglobin (g/dL)	Body temperature (°C)	Note
Osarfo, 2017 [52]	ASAQ	205	15-19 (20%), 20-24 (27%), 25-29 (27%), ≥30 (26%)	NA	22.3 (95% CI: 21.6-22.9)	0 (26%), 1 (22%), ≥2 (51%)	NA	238‡ (95%CI: 199-285)	10.1 (95%CI: 9.9-10.2)	Asymptomatic	Women with positive HIV status were excluded.
	DP	212	15-19 (14%), 20-24 (36%), 25-29 (32%), ≥30 (18%)		22.7 (95%CI: 22.1-23.4)	0 (35%), 1 (22%), ≥2 (43%)		236‡ (95%CI: 193-289)	10.0 (95%CI: 9.8-10.2)		
Onyamboko, 2015 [53]	AL3<br>AL5	48<br>48	NA	NA	NA	NA	NA	NA	NA	NA	Conference abstract
Ukah, 2015 [54]	ASAQ	75	29.2 ± 4.8 (20–42)	NA	28.5 ± 6.8 (14–40)	NA	1* (0–4)	13174 ± 12445	Hct 32.3 ± 2.4 %	Fever 60%<br>Fever 53%	HIV-positive cases: 1/75 in ASAQ, 5/75 in AL..
	AL	75	29.4 ± 4.6 (18–41)		27.2 ± 7.3 (14–40)		0* (0–4)	12484 ± 11051	Hct 32.2 ± 2.3 %		
Iribhogbe, 2017a [55]	AL<br>ASAQ	40<br>40	31.0 ± 0.5<br>27.2 ± 0.5	68.2 ± 0.3<br>69.0 ± 0.9	27.9 ± 0.3<br>31.3 ± 0.0	3.4 ± 0.7<br>2.9 ± 0.1	4.3 ± 0.6<br>3.7 ± 0.1	4884 ±386.6<br>3634 ± 537	NA	38.5 ± 0.1<br>38.1 ±0.1	
McGready, 2003a [58]	AAP	24	20* (16–37)	50* (39–64)	28.5* (19.1–35.9)	2* (1–7)	1* (0–4)	3467‡ (33–138038)	NA	37.6* (36.0–40.0)	
Adam, 2012 [59]	DP	12	26.2 ± 8.7	61.3 ± 10.1	32.0 (15.0–40.0)	NA	NA	12642‡ (1716–89700)	9.0 ± 1.0	NA	
Onyamboko, 2011 [60]	ASSP<br>EGA 22–26w<br>EGA 32–36w	13<br>13	21.7 ± 2.7<br>26.6 ± 4.4	55.5 ± 8.3<br>63.0 ± 7.2	22.4 ± 0.8<br>33.2 ± 1.4	NA	0* (0–1)<br>1* (0–4)	528* [372–842]	Hct 30.0* [30.0–30.0] %<br>Hct 32.0* [31.0–33.0] %	36.5* [36.1–36.7]<br>36.4* [36.2–36.5]	All HIV negative. Including 2 women with submicroscopic parasitaemia.
McGready, 2012 [61]	AS (iv on day0)<br>AS (iv on day1)	10<br>10	24* (17–40)<br>32* (22–40)	48* (40–59)<br>50* (40–64)	24.8* (17.1–24.2)<br>25.5* (14.0–37.4)	1* (1–6)<br>4* (1–8)	0* (0–5)<br>2* (0–6)	7933‡ (575–63096)<br>2864 ‡ (129–51286)	Hct 30* (25–46) %<br>Hct 30* (27–34) %	37.8* (36.0–40.0)<br>37.1* (36.1–39.0)	
Rijken, 2011 [62]	DP	24	28.1 ± 8.0	49 ± 6	24.6 ± 5.9	3* (1–9)	2* (0–8)	7349.06‡ (96–176594)	Hct 31.0 ± 4.5 %	37.7 ± 0.9	

Additional file 7 continued.

Study [reference]	Treatment group	N	Age (year)	Weight (kg)	Gestational age at malaria (week)	Gravidity	Parity	Parasitaemia (/µL)	Haemoglobin (g/dL)	Body temperature (°C)	Note
Valea, 2014 [63]	ASMQ	24	23.6 ± 6.1	52.0* (46–69.5)	2nd tri (50%)<br>3rd tri (50%)	NA	1 (42%)<br>2 (33%)<br>3 (25%)	1029‡ (95%CI 455–2327)	9.0 ± 1.1	36.9 ± 0.4	
Juma, 2014 [64]	AL	45	NA	NA	NA	NA	NA	NA	NA	NA	Conference abstract and registry information
Mosha, 2014 [65]	AL	33	25* (18–41)	52* (40–80)	27* (14–37)	NA	NA	25280* (560–198080)	10.2* (7.1–13.3)	37.1* (36.0–39)	
Nyunt, 2016 [66]	AL	30	25* (18–39)	59.4* (44.5–81.1)	28* (14–34)	NA	NA	13227‡ (95%CI 7728–22639)	10.5* (7.6–13.1)	NA	All HIV negative.
Mutagonda, 2017 [68]	AL	92	23* (15–41)	NA	2nd tri (61%)<br>3rd tri (39%)	1 (46%)<br>2 (19%)<br>≥3 (36%)	NA	2700* (400–72500)	NA	NA	
Adam, 2004b [69]	Q	26	26.2 ± 3.5	65.8 ± 4.6	8.5 ± 0.9	NA	2.8 ± 2.6	5856 ± 1652	9.2 ± 1.3	38.2 ± 0.6	
Adam, 2004c [70]	AM	28	27.1 ± 5.8	65.8 ± 6.4	27 ± 7.3	NA	1* (0–8)	8468.54‡ (525–35000)	8.3 ± 0.9	38.6 ± 1.1	
Adegnika, 2005 [71]	Q Recurrence	20	20 ± 4	54 ± 6	25.5 ± 5.7	2.2 (1–5)	0.8 (0–3)	2104‡ (125–240000)	9.0 ± 1.2	36.8 ± 0.5	
	Q No recurrence	30	23 ± 6	61 ± 8	24.5 ± 6.3	3.2 (1–13)	1.6 (0–9)	695‡ (12–19800)	9.5 ± 1.1	36.8 ± 0.4	
Adam, 2006 [72]	ASSP	32	29.4 ± 4.3	68.7 ± 12.7	29.7 ± 8.4	NA	3.2 ± 1.8	7762‡ (3800–15250)	9.8 ± 1.4	38.1 ± 0.6	
Ndiaye, 2011 [73]	ASAQ	28	NA	NA	NA	NA	NA	9.91 ± 1.24‡	NA	NA	Conference abstract. Parasitaemia is shown in log scale.
Iribhogbe, 2017b [74]	ASAQ	50	NA	NA	NA	NA	NA	5721.3 ± 287.0	NA	100%	Demographic information was shown together with non-malaria pregnant women.

Additional file 7 continued.

Study [reference]	Treatment group	N	Age (year)	Weight (kg)	Gestational age at malaria (week)	Gravidity	Parity	Parasitaemia (/μL)	Haemoglobin (g/dL)	Body temperature (°C)	Note
McGready, 1998a [75]	Artemisinins Retreatment<br>Hyperparasitaemia<br>Inadvertent use	61<br>13<br>16	25 (17–38)<br>22 (16–32)<br>20 (15–40)	NA	1st (28%)<br>2nd–3rd (82%)<br>1st (88%)<br>2nd–3rd (12%)	NA	NA	814* (16–130385)<br>115484* (960–312924)<br>20788* (50–394881)	Anaemia 72%<br>Anaemia 50%<br>Anaemia 29%	Fever 16%<br>Fever 23%<br>Fever 67%	Gestational age was reported for combined group of retreatment and hyperparasitaemia. Percentage of anaemia was reported but without definition.
McGready, 1998b [76]	Q<br>Primary treatment<br>Retreatment	113<br>91	24.1 ± 5.8<br>24.5 ± 6	47 ± 5.9<br>49.6 ± 7	14.7 ± 7.3<br>22.6 ± 8	1 (37%)<br>2–3 (31%)<br>>3 (32%)<br>1 (35%)<br>2–3 (35%)<br>>3 (31%)	NA	852‡ (16–2000332)<br>1078‡ (4–53719)	Hct 29.5 ± 5.3%<br>Hct 28.2 ± 5%	36.7 ± 1<br>36.6 ± 0.82	Including multiple episodes from a single patient.
McGready, 2001b [77]	Artemisinins<br>Primary treatment<br>Retreatment	229<br>310	24.8 ± 6.4 (14–48)	48.8 ± 6.7 (32–78)	24.6 ± 9.3 (3–40)	3* (1–17)	1* (0–14)	1023‡ (16–131825)<br>1412‡ (16–125892)	Hct 28.9 ± 5.1 %<br>Hct 28.1 ± 5.1 %	Fever 18%	Including 55 cases of severe or hyperparasitaemia. Parasitaemia of uncomplicated cases are shown.
Laochan, 2015 [78]	AS<br>AAP<br>AC<br>AL<br>ASMQ<br>DP<br>Q<br>QC<br>MQ	151<br>11<br>143<br>43<br>5<br>29<br>476<br>29<br>22	24.5* (14–45)	47* (30–78)	21* (4–37)<br>23* (18–32)<br>23* (1–39)<br>23* (13–36)<br>28* (-0.4–39)<br>20* (8–34)<br>17* (3–36)<br>10* (4–24)<br>26* (12–38)	NA	NA	3216* (16.4–4768)	Hct 31* (12–48) %	36.8* (35.0–40.4)	Summary of all 700 pregnant women included in the analysis except gestational age.
McGready, 2002 [79]	Q	204	24 ± 7 (14–42)	45.4 ± 5.9 (35–66)	7.9 ± 2.8 (1.0–12.8)	2* (1–13)	1* (0–10)	1230‡ (15.8–138038)	Anaemia[a] 29%	Fever 18%	
McGready, 2003b [80]	AAP	27	25* (16–38)	NA	28.2* (-1.6–37.3)	3* (1–7)	1* (0–6)	2206‡ (16–309030)	Hct 29.3 ± 5.1 %	Fever 7%	
Villegas, 2005 [81]	ASMQ	27	NA	NA	NA	NA	NA	NA	NA	NA	Conference abstract

Additional file 7 continued.

Study [reference]	Treatment group	N	Age (year)	Weight (kg)	Gestational age at malaria (week)	Gravidity	Parity	Parasitaemia (/µL)	Haemoglobin (g/dL)	Body temperature (°C)	Note
Rijken, 2008 [82]	DP	62	24* (16–39)	48 ± 6 (36–60)	23.7 ± 8.2 (9.2–39.1)	2* (1–12)	1* (0–9)	1951‡ (12–85114)	Hct 29.9 ± 4.3 % (18–37)	36.6 ± 1.0 (35.1–40.0)	62 episodes in 50 patients.
Rulisa, 2012 [83]	AL	(1072)	26.0* (17–48)	NA	25.8* (95%CI 25.3–26.4)	2* (1–12)	1* (0–10)	NA	NA	NA	Including patients with clinical diagnosis. The number of patients with confirmed parasitaemia is not specified.
Kalilani, 2013 [84, 85]	AL	56	NA	NA	NA	NA	NA	NA	NA	NA	Only summary of the whole cohort including women without malaria was reported.

AAP: artesunate-atovaquone-proguanil, AC: artesunate-clindamycin, AL: artemether-lumefantrine, AQ: amodiaquine, AS: artesunate, CHQ: chloroquine, CI: confidence interval, DP: dihydroartemisinin-piperaquine, EGA: estimated gestational age, Hct: haematocrit, IQR: inter-quartile range. MQ: mefloquine, N: number of pregnant women who were assessed, NA: data not available, Q: quinine, QC: quinine-clindamycin, SP: sulfadoxine-pyrimethamine, tri: trimester.

Figures are shown in mean (± standard deviation) unless indicated otherwise. *: median. ‡: geometric mean. Rages are shown in ( ) and inter-quartile ranges are shown in [ ]. Gestational age at malaria, parasitaemia and haemoglobin were shown in week, /µL and g/dL, respectively, unless indicated otherwise. Gestational age, gravidity and parity are shown as category (%) depending on the reporting.

[a]: anaemia was defined as haematocrit <30%.
