# Supplementary material for: Systematic literature review and meta-analysis of the efficacy of artemisinin-based and quinine-based treatments for uncomplicated falciparum malaria in pregnancy: methodological challenges
Source: Malar J. 2017 Dec 13;16:488. doi: 10.1186/s12936-017-2135-y (PMC5729448; doi:10.1186/s12936-017-2135-y)
Supplement: Supplementary file 8 — Additional file 8. Summary of gametocyte carriage. [file 12936_2017_2135_MOESM8_ESM.pdf]

Additional file 8. Summary of gametocyte carriage

Gametocytes were assessed in 16 studies (33%, 16/48). ABT was more effective in clearing and also suppressing the development of gametocytaemia than non-ABT. AL showed better gametocyte clearance on day 2 and day 7 than quinine [48]. The proportion of gametocytaemia development after treatment was lower in artesunate-atovaquone-proguanil (AAP) than in quinine (5% and 29%, respectively) [42] and the prevalence of gametocytes on day 14 was lower in ASAQ than in SP (5% and 31%, respectively) [46].

Gametocyte carriage defined by the number of weeks with positive gametocytaemia divided by the total number of weeks of follow-up was 2–26.8/1000 person-week in ABT and 39–92.7/1000 person-week in QBT in studies conducted on the Thailand-Myanmar border (Table in Additional file 8). This difference was significant in three RCTs directly comparing ABT and QBT [40-42]. Gametocyte carriage was not different between the different ABT in two RCTs [45,50].

Table Gametocyte carriage following the quinine-based and artemisinin-based treatments.

**Study [reference]**	**Treatment**	**Subgroup**	**Gametocyte carriage (person-week/1000)**
McGready, 1998b [76]	Q	Retreatment	92.7 (95%CI 57.4–128)
McGready, 1998b [76]	Q	Primary treatment	79.8 (95%CI 51.5–108.1)
McGready, 2000 [40]	Q		46.9 (95%CI 26–78)
McGready, 2002 [79]	Q		42.5 (95%CI 27.8–62.1)
McGready, 2005 [50]	Q		49 (95%CI 26–89)
McGready, 2001a [41]	QC		39 (95%CI 21–66)
McGready, 2001b [77]	Artemisinins	Retreatment	26.8 (95%CI 18.5–37.4)
Rijken, 2008 [82]	DP		11.7 (95%CI 3.0–36.7)
McGready, 2001b [77]	Artemisinins	Primary treatment	11.4 (95%CI 5.5–20.8)
McGready, 2008 [45]	AS		10 (95%CI 4–21)
McGready, 2005 [42]	AAP		6 (95%CI 1–25)
McGready, 2001a [41]	AS		3 (95%CI 0–19)
McGready, 2000 [40]	ASMQ		2.3 (95%CI 0–11)
McGready, 2008 [45]	AL		2 (95%CI 0–10)

AAP: artesunate-atovaquone-proguanil, AL: artemether-lumefantrine, AS: artesunate, CI: confidence interval, DP: dihydroartemisinin-piperaquine, MQ: mefloquine, Q: quinine, QC: quinine-clindamycin.
