# Supplementary material for: Systematic literature review and meta-analysis of the efficacy of artemisinin-based and quinine-based treatments for uncomplicated falciparum malaria in pregnancy: methodological challenges
Source: Malar J. 2017 Dec 13;16:488. doi: 10.1186/s12936-017-2135-y (PMC5729448; doi:10.1186/s12936-017-2135-y)
Supplement: Supplementary file 9 — Additional file 9. Funnel plots by quinine-based and artemisinin-based treatments. [file 12936_2017_2135_MOESM9_ESM.pdf]

Quinine-based treatment

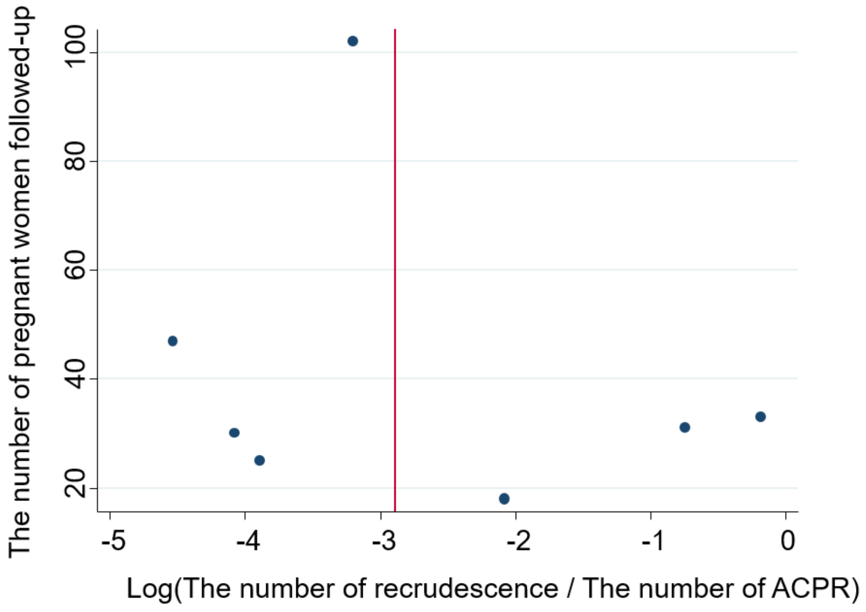

Artemisinin-based treatment

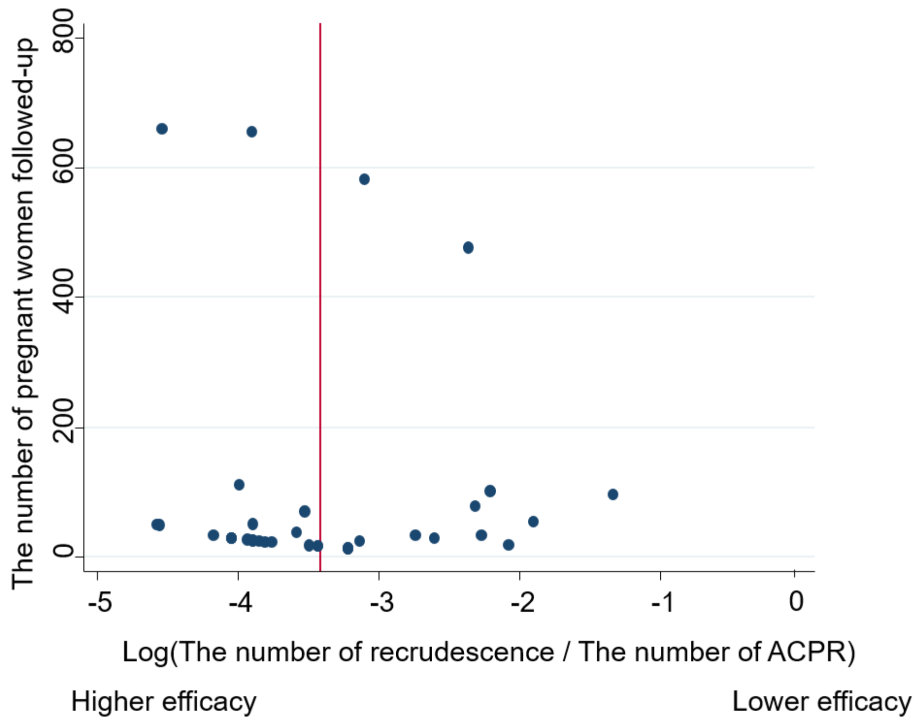

Additional file 9. Funnel plots by quinine-based and artemisinin-based treatments. Log odds (the number of PCR-confirmed recrudescence / the number of PCR-corrected ACPR) was plotted on the x-axis against the number of pregnant women followed-up on the y-axis. Each study arm was plotted once using the treatment outcome of the longest follow-up. Continuity correction was made for the studies without treatment failure by adding 0.5. The vertical line shows the average of the log odds weighted by the study size.
