# Supplementary material for: Systematic literature review and meta-analysis of the efficacy of artemisinin-based and quinine-based treatments for uncomplicated falciparum malaria in pregnancy: methodological challenges
Source: Malar J. 2017 Dec 13;16:488. doi: 10.1186/s12936-017-2135-y (PMC5729448; doi:10.1186/s12936-017-2135-y)

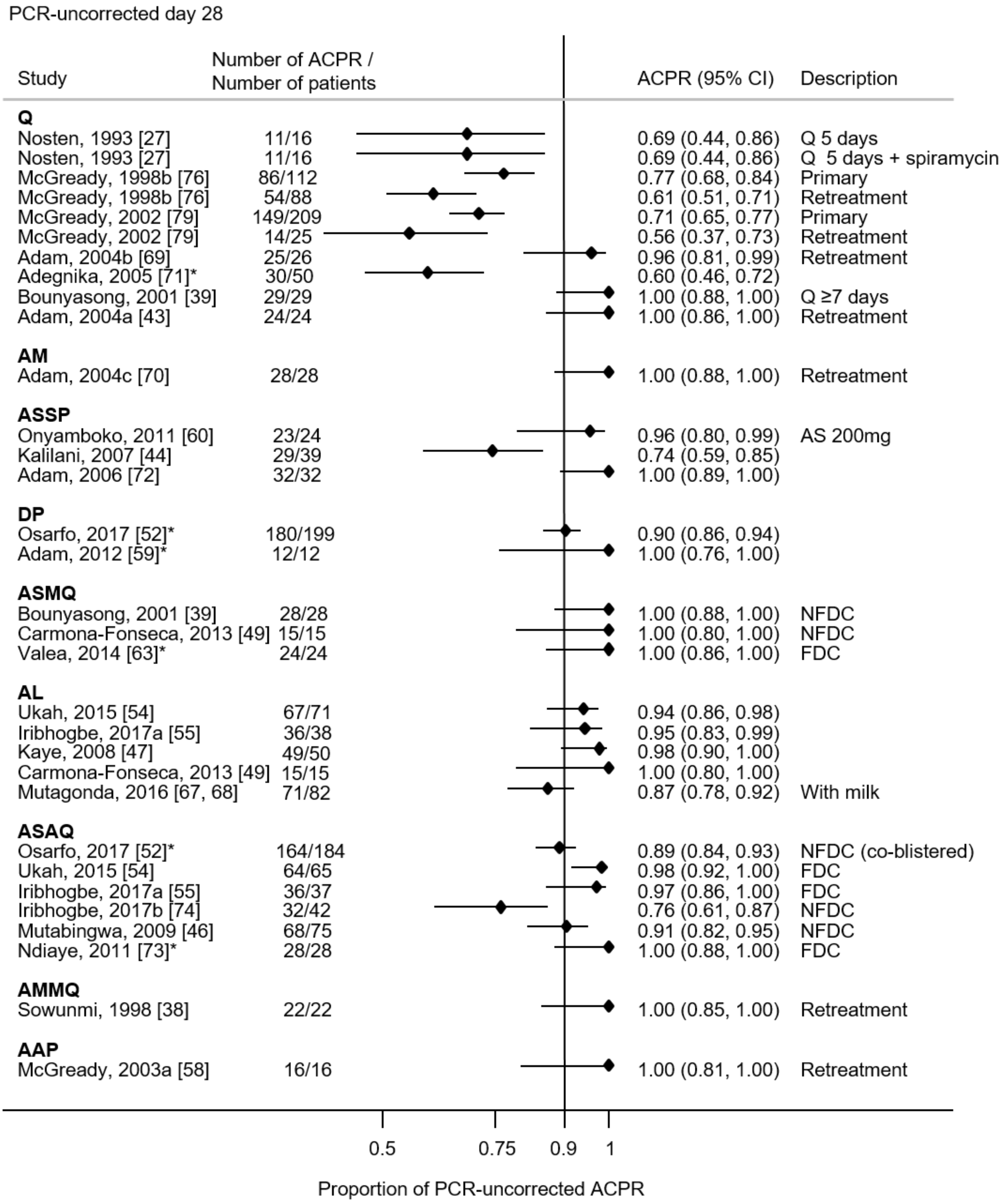

Additional file 10 The PCR-uncorrected proportional adequate clinical and parasitological response (ACPR). ACPR with 95% confidence intervals are shown by treatment group and the duration of follow-up. *: ACPR at later follow-up day is available. AAP: artesunate + atovaquone-proguanil, AC: artesunate + clindamycin, AL: artemether-lumefantrine, AM: artemether, AS: artesunate, AQ: amodiaquine, DP: dihydroartemisinin-piperaquine, FDC: fixed dose combination, MQ: mefloquine, NFDC: non-fixed dose combination, Q: quinine, QC: quinine + clindamycin, SP: sulfadoxine-pyrimethamine.

Additional file continued.

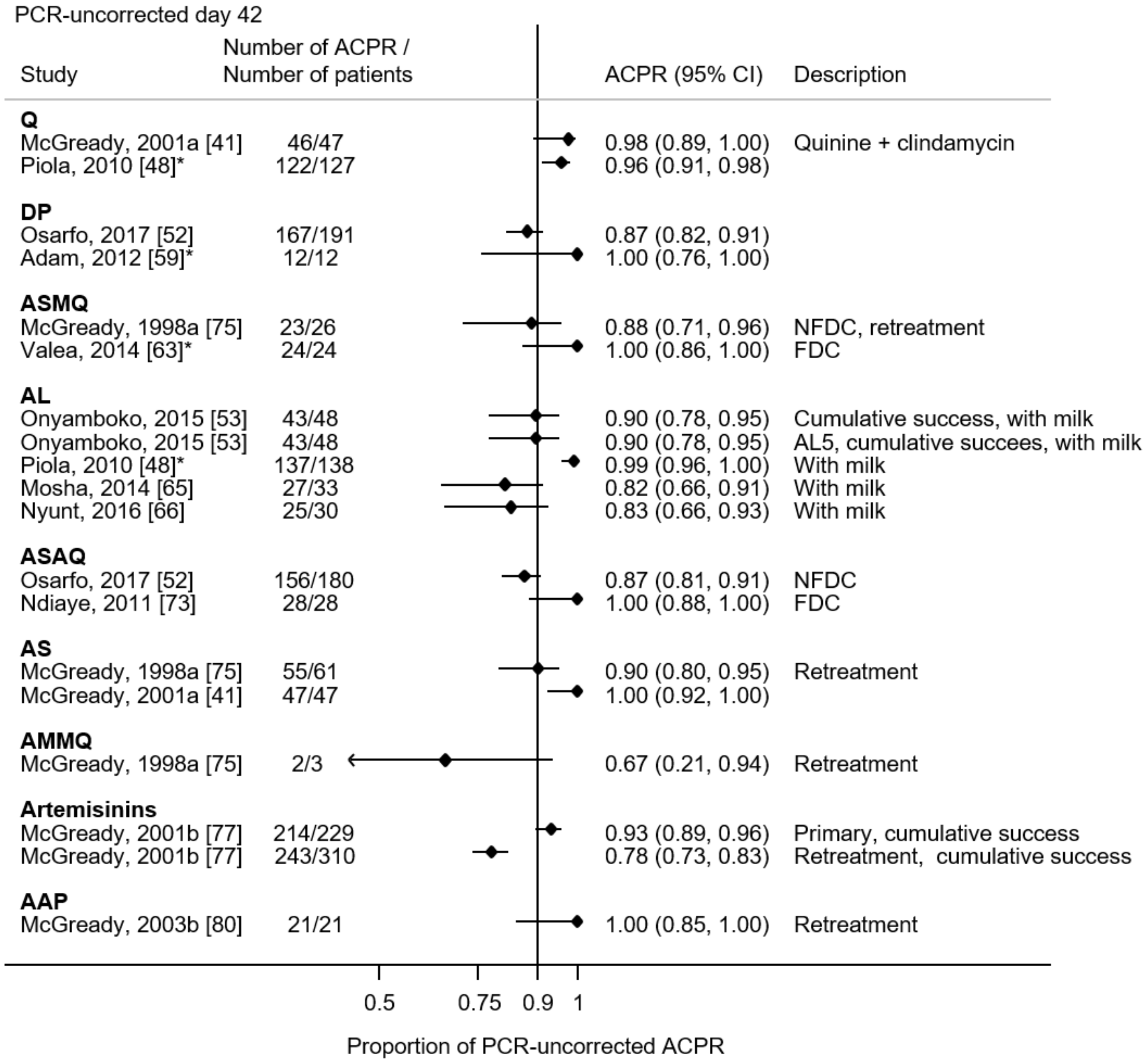

Additional file continued.

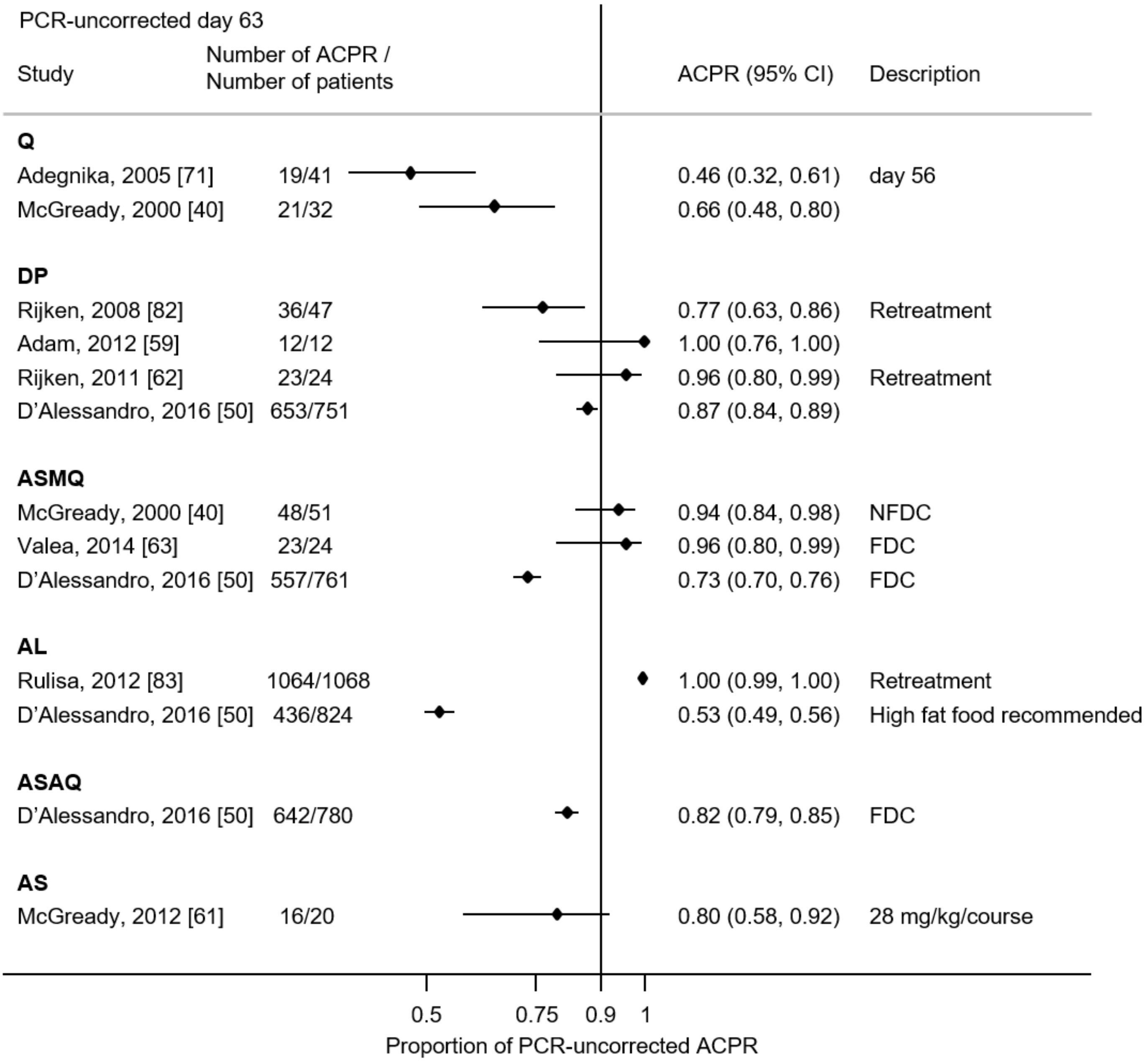

Additional file continued.

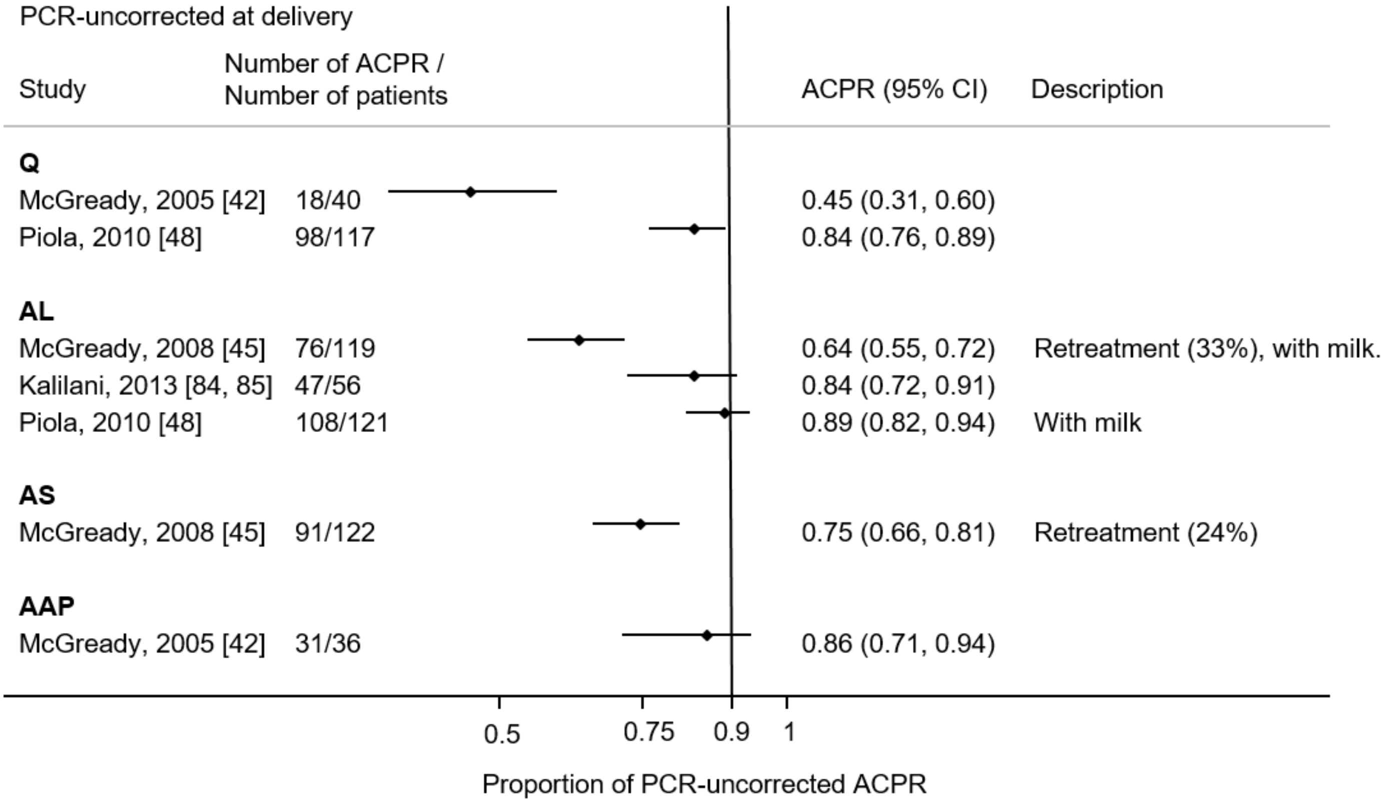

Supplement: Supplementary file 10 — Additional file 10. The PCR-uncorrected proportional adequate clinical and parasitological response (ACPR). [file 12936_2017_2135_MOESM10_ESM.pdf]
