# Supplementary material for: Systematic literature review and meta-analysis of the efficacy of artemisinin-based and quinine-based treatments for uncomplicated falciparum malaria in pregnancy: methodological challenges
Source: Malar J. 2017 Dec 13;16:488. doi: 10.1186/s12936-017-2135-y (PMC5729448; doi:10.1186/s12936-017-2135-y)
Supplement: Supplementary file 11 — Additional file 11. Summary of evidence profile comparing artemisinin-based and quinine-based treatment. [file 12936_2017_2135_MOESM11_ESM.pdf]

Additional file 11 Summary of evidence profile comparing artemisinin-based and quinine-based treatment

Quality assessment							№ of patients		Effect		Quality	Importance
**№ of studies**	**Study design**	**Risk of bias**	**Inconsistency**	**Indirectness**	**Imprecision**	**Other considerations**	**Artemisinin-based treatment**	**Quinine-based treatment**	**Relative (95% CI)**	**Absolute (95% CI)**		
PCR-corrected treatment failure (follow up: 28 days)												
1 [39]	randomised trials	very serious [a,b,c]	not serious	not serious	very serious [d,e]		0/28	0/29	**Rate ratio 1.0** (0.0 to 50.4)	-	Very Low	CRITICAL
PCR-corrected treatment failure (follow up: 42 days)												
2 [41, 48]	randomised trials	serious [a,b]	not serious	not serious	serious [d]		1/185 (0.5%)	3/170 (1.8%)	**RR 0.39** (0.06 to 2.62)	**11 fewer per 1,000** (from 17 fewer to 29 more)	Low	CRITICAL
PCR-corrected treatment failure (follow up: 63 days)												
1 [40]	randomised trials	serious [a,b]	not serious	not serious	not serious	Large effect	0/63 (0.0%)	9/40 (22.5%)	**RR 0.03** (0.00 to 0.56)	**218 fewer per 1,000** (from -- to 99 fewer)	Moderate	CRITICAL
PCR-corrected treatment failure (follow up: delivery)												
2 [42, 48]	randomised trials	serious [a,b]	not serious	not serious	not serious	Large effect	4/143 (2.8%)	19/135 (14.1%)	**RR 0.20** (0.07 to 0.58)	**113 fewer per 1,000** (from 59 fewer to 131 fewer)	Moderate	CRITICAL

Quality assessment							№ of patients		Effect		Quality	Importance
№ of studies	Study design	Risk of bias	Inconsistency	Indirectness	Imprecision	Other considerations	Artemisinin-based treatment	Quinine-based treatment	Relative (95% CI)	Absolute (95% CI)		
Placental malaria												
1 [48]	randomised trials	serious [a]	not serious	serious [f]	serious [d]		5/110 (4.5%)	5/102 (4.9%)	**RR 0.93** (0.28 to 3.11)	**3 fewer per 1,000** (from 35 fewer to 103 more)	Very low	IMPORTANT
Gametocyte carriage (assessed with: person-week)												
3 [40-42]	randomised trials	serious [a,b]	not serious	not serious	not serious		A significant difference was reported in all three RCTs. Q(46.9 (95%CI 26-78)) vs ASMQ (2.3 (95%CI 0-11)); QC (39 (95%CI 21-66)) vs AS (3 (95%CI 0-19)); Q (42.5 (95%CI 27.8-62.1)) vs AAP (6 (95%CI 1-25))				Moderate	IMPORTANT
Tinnitus												
5 [39-42, 48]	randomised trials	serious [a,b,c]	not serious	not serious	not serious	Large effect	42/303 (13.9%)	221/298 (74.2%)	**RR 0.21** (0.05 to 0.83)	**586 fewer per 1,000** (from 126 fewer to 705 fewer)	High	IMPORTANT
Vomiting												
5 [39-42, 48]	randomised trials	very serious [a,b,c]	serious [g]	not serious	not serious		39/330 (11.8%)	78/316 (24.7%)	**RR 0.49** (0.29 to 0.84)	**126 fewer per 1,000** (from 39 fewer to 175 fewer)	Moderate	IMPORTANT

Quality assessment							№ of patients		Effect		Quality	Importance
**№ of studies**	**Study design**	**Risk of bias**	**Inconsistency**	**Indirectness**	**Imprecision**	**Other considerations**	**Artemisinin-based treatment**	**Quinine-based treatment**	**Relative (95% CI)**	**Absolute (95% CI)**		
Dizziness												
3 [40, 41, 48]	randomised trials	serious [a,b]	serious [g]	not serious	not serious		33/198 (16.7%)	49/202 (24.3%)	**RR 0.66** (0.44 to 0.98)	**82 fewer per 1,000** (from 5 fewer to 136 fewer)	Moderate	IMPORTANT
Hypoglycemia												
1 [39]	randomised trials	very serious [a,b,c]	not serious	not serious	not serious	Large effect	3/28 (10.7%)	21/29 (72.4%)	**RR 0.15** (0.05 to 0.44)	**616 fewer per 1,000** (from 406 fewer to 688 fewer)	Low	CRITICAL
Haemoglobin on day 7												
3 [40, 41, 48]	randomised trials	serious [a]	not serious	not serious	not serious		There was a significant difference in the changes of haemoglobin level between AL (-0.5g/dL) and quinine (+0.3g/dL). The mean haematocrit on day 7 was lower in ASMQ (27.5±3.9%) than in quinine (29.5±4.4%). The mean haematocrit on day 7 was lower in artesunate than in quinine + clindamycin (27.6 ± 4.2% and 29.6 ± 4.1%, respectively).				Low	IMPORTANT
Pregnancy loss												
5 [39-42, 48]	randomised trials	not serious	not serious	very serious [h]	very serious [d]		9/297 (3.0%)	8/319 (2.5%)	**RR 1.04** (0.41 to 2.66)	**1 more per 1,000** (from 15 fewer to 42 more)	Low	CRITICAL

Quality assessment							№ of patients		Effect		Quality	Importance
№ of studies	Study design	Risk of bias	Inconsistency	Indirectness	Imprecision	Other considerations	Artemisinin-based treatment	Quinine-based treatment	Relative (95% CI)	Absolute (95% CI)		
Congenital abnormality												
5 [39-42, 48]	randomised trials	not serious	not serious	very serious [h]	very serious [d]		4/297 (1.3%)	3/319 (0.9%)	**RR 1.39** (0.32 to 6.14)	**4 more per 1,000** (from 6 fewer to 48 more)	Low	CRITICAL

CI: Confidence interval; **RR:** Risk ratio by a random effects model.

a. Open-label studies b. Allocation concealment is not clear. c. Randomisation is not clear. d. Wide CI including 1.0. e. Sample size was not calculated beforehand. f. It is difficult to show which malaria episode in pregnancy is the cause of the placental malaria. g. The overall prevalence was very different. h. This outcome can be caused by many factors.

High	Further research is very unlikely to change our confidence in the estimate of effect.
Moderate	Further research is likely to have an important impact on our confidence in the estimate of effect and may change the estimate.
Low	Further research is very likely to have an important impact on our confidence in the estimate of effect and is likely to change the estimate.
Very low	Any estimate of effect is very uncertain.
