# Supplementary material for: Systematic literature review and meta-analysis of the efficacy of artemisinin-based and quinine-based treatments for uncomplicated falciparum malaria in pregnancy: methodological challenges
Source: Malar J. 2017 Dec 13;16:488. doi: 10.1186/s12936-017-2135-y (PMC5729448; doi:10.1186/s12936-017-2135-y)
Supplement: Supplementary file 12 — Additional file 12. Funnel plot of odds ratio of PCR-corrected treatment failure comparing quinine-based and artemisinin-based treatments. [file 12936_2017_2135_MOESM12_ESM.pdf]

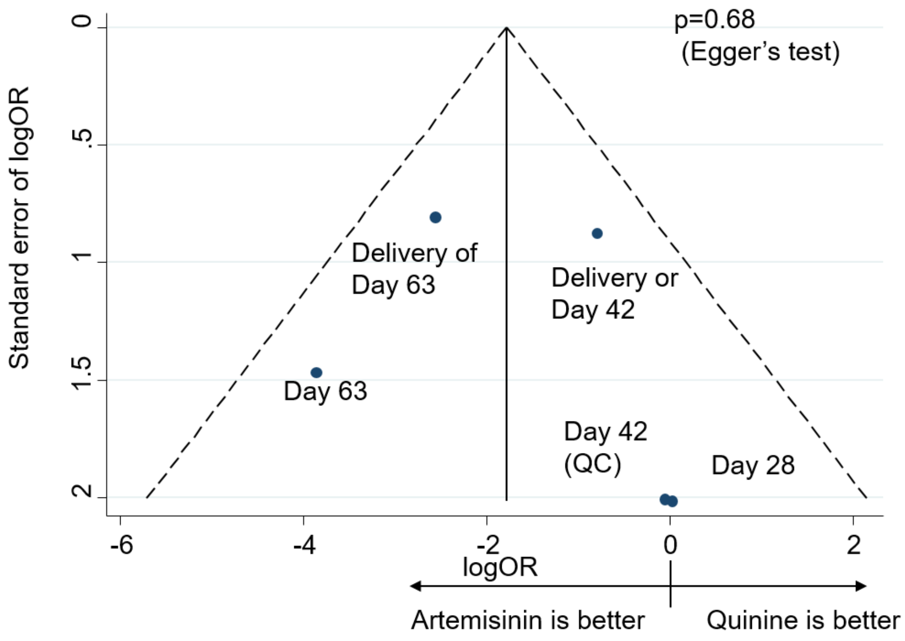

Additional file 12 Funnel plot of odds ratio (OR) of PCR-corrected treatment failure comparing quinine-based and artemisinin-based treatments. Continuity correction was made for the studies without treatment failure by adding 0.5. QC: quinine + clindamycin.
