# Supplementary material for: Systematic literature review and meta-analysis of the efficacy of artemisinin-based and quinine-based treatments for uncomplicated falciparum malaria in pregnancy: methodological challenges
Source: Malar J. 2017 Dec 13;16:488. doi: 10.1186/s12936-017-2135-y (PMC5729448; doi:10.1186/s12936-017-2135-y)
Supplement: Supplementary file 13 — Additional file 13. The methodology of assessing placental malaria. [file 12936_2017_2135_MOESM13_ESM.pdf]

Additional file 13. The methodology of assessing placental malaria

Study [reference]	Method of evaluating placental malaria
Bounyasong, 2001 [39]	Calcification of placenta
	Pathological examination of placenta
Kalilani, 2007 [44]	Placental blood and cord blood thick smears
	Placental histopathology (Rogerson method)
McGready, 2008 [45]	Placental blood (smear and PCR)
	Cord blood (smear and PCR)
Mutabingwa, 2009 [46]*	Placental blood smear
	Cord blood smear
Piola, 2010 [48]	Placental histopathology (Rogerson method)
	Placental blood (smear and PCR)
	Placental crush smear
	Cord blood (smear and PCR)
D'Alessandro, 2016 [50]	Placental biopsy Giemsa stain.
	Placental histopathology (Ismail method)
Osarfo, 2017 [52]	Placental parasitaemia
	Cord blood parasitaemia
Onyamboko, 2015 [53]	Yes (details not available)
NCT01054248 [57]	Cord blood (smear and PCR)
	Placenta (smear and PCR)
	Placenta histopathology
Rijken, 2011 [62]	Placental smear
Adegnika, 2005 [71]	Placental thick blood smear
Ndiaye, 2011 [73]	Placental blood
	Cord blood
McGready, 2003b [80]	Placental smear
Kalilani, 2013 [84, 85]	Placental histopathology (Presence of parasites or pigment were regarded as positive.)
	Placental blood quantitative PCR if histopathology is positive.

PCR: polymerase chain reaction.
* Planned in the protocol but not reported.
